# Characterizing Movement Fluency in Musical Performance: Toward a Generic Measure for Technology Enhanced Learning

**DOI:** 10.3389/fpsyg.2019.00084

**Published:** 2019-02-04

**Authors:** Victor Gonzalez-Sanchez, Sofia Dahl, Johannes Lunde Hatfield, Rolf Inge Godøy

**Affiliations:** ^1^RITMO Centre for Interdisciplinary Studies in Rhythm, Time and Motion, Department of Musicology, University of Oslo, Oslo, Norway; ^2^Department of Architecture, Design and Media Technology, Aalborg University, Copenhagen, Denmark; ^3^Department of Music Education and Music Therapy, Norwegian Academy of Music, Oslo, Norway

**Keywords:** motor control, music performance, coarticulation, EMG, motion capture, phase transition, smoothness

## Abstract

Virtuosity in music performance is often associated with fast, precise, and efficient sound-producing movements. The generation of such highly skilled movements involves complex joint and muscle control by the central nervous system, and depends on the ability to anticipate, segment, and coarticulate motor elements, all within the biomechanical constraints of the human body. When successful, such motor skill should lead to what we characterize as fluency in musical performance. Detecting typical features of fluency could be very useful for technology-enhanced learning systems, assisting and supporting students during their individual practice sessions by giving feedback and helping them to adopt sustainable movement patterns. In this study, we propose to assess fluency in musical performance as the ability to smoothly and efficiently coordinate while accurately performing slow, transitionary, and rapid movements. To this end, the movements of three cello players and three drummers at different levels of skill were recorded with an optical motion capture system, while a wireless electromyography (EMG) system recorded the corresponding muscle activity from relevant landmarks. We analyzed the kinematic and coarticulation characteristics of these recordings separately and then propose a combined model of fluency in musical performance predicting music sophistication. Results suggest that expert performers' movements are characterized by consistently smooth strokes and scaling of muscle phasic coactivation. The explored model of fluency as a function of movement smoothness and coarticulation patterns was shown to be limited by the sample size, but it serves as a proof of concept. Results from this study show the potential of a technology-enhanced objective measure of fluency in musical performance, which could lead to improved practices for aspiring musicians, instructors, and researchers.

## 1. Introduction

The movements of an expert musician typically appear as smooth and graceful, with highly complex tasks seeming to come at a minimum of effort (Altenmüller and Schneider, 2009; Jørgensen and Hallam, 2008). This quality of smoothness and flow of movement can be defined as *fluency* (Whiting et al., 1987; Kerr et al., 2013). In the case of music performance, fluency relates to the perceived ability to perform complex sound-producing movements with both accuracy and efficiency. Fluency is often focused on by practitioners, teachers and physiotherapists to evaluate whether the movement patterns of a student or patient are developing in the right direction (Van Dokkum et al., 2012; Kerr et al., 2013; Volta et al., 2018). In the context of technology enhanced learning, a way to accurately quantify and measure such fluency would be very useful, for instance in automated systems for feedback to music students. The right type of feedback is most relevant when students are practicing at home, without the presence of a teacher to guide them. And since fluency is relevant for many kinds of motor skills, implementing this in systems for home practice could make it useful for several different kinds of instrument practice.

The foundations and handcraft that enable expertise and fluency is particularly interesting from an educational perspective. Metcalf et al. (2014) proposed a model of skill acquisition as a spectrum for indexing the level of complex hand dexterity. At the lowest end of the defined spectrum is the impaired, while higher levels span the progression of expertise from novice to practitioner, expert, and finally: virtuoso. Although intended for hand dexterity, the proposed spectrum of Metcalf et al. (2014) is a useful model for discussing skill acquisition in general, and for music skill in particular. The goal from a didactic perspective is to enable a music student to progress from novice to expert. And importantly, the training should also avoid factors leading to impairment such as misuse, excessive overuse, and straining repetition of movements (Metcalf et al., 2014). Since music training often has a particular focus on the produced sound and timing patterns rather than the movements, there is a potential risk that students develop less optimal movement patterns (Visentin et al., 2008). This, in combination with the often extensive training needed for musical performance, can create problems later on in the career path of musicians, leading to strain, pain, or even development of dysfunctional movement patterns such as those seen in focal dystonia (Altenmüller and Jabusch, 2010).

Performance achievement in various fields can be related to the amount and quality of practice (Ericsson, 2013). Researchers studying skill acquisition of top chess players, medical surgeons, top-athletes, and musicians have generally found that the ways in which the experts in each domain practice are highly similar (Ericsson et al., 2018). High levels of performance achievement in various fields can primarily be explained by the amount and quality of practice, implying that so-called talent is over-rated. According to Ericsson (2014), Ericsson and Pool (2016), effective skill acquisition is based on the extent to which we: (a) identify and concentrate on the relevant aspects of performance, (b) get relevant and immediate feedback, and (c) gradually refine skill through repetition and problem solving. Hence, adequate feedback on movement patterns during musical performance would help to guide the students along their learning path.

In our present exploratory study, we aim at characterizing fluency in music performance as the ability to efficiently make smooth and precise movements, and to do so both slowly and rapidly. The objective is to explore movement fluency by quantifying end-effector movement smoothness and assessing motor coordination patterns through principal component analysis. As a proof of concept that the approach could be valid for different kinds of movements, we recorded and analyzed movements and relevant muscle activity of cello players and drummers of varied expertise. The paper is organized as follows: In section 2, we give an overview of the characteristics of musicians' movement skills and different approaches in technology assisted teaching of these skills. In section 3, we define how fluency can be measured, and how generic movement types making comparisons across performances on different instruments are possible. In sections 4, 5, and 6, we outline the details and results of of our exploratory study. Finally, in section 7, we discuss these results within the framework of music skills acquisition and music practice, aimed at contributing to the development of updated teaching, assessment, and scientific research methods.

## 2. Achieving Movement Fluency

For several decades, we have seen the publication of a number of works presenting methods for proper instrumental practice (e.g., Auer, 1960; Leimer and Gieseking, 1972), with an increasing number of studies investigating musicians' motor skill. The focus of these studies have primarily been on piano performance (e.g., Ortmann, 1962; Halsband et al., 1994; Engel et al., 1997; Jabusch et al., 2009; Furuya and Altenmüller, 2013; Goebl and Palmer, 2013; Metcalf et al., 2014; Furuya et al., 2015; Goebl, 2018), but a number of works also exist for bowed instruments such as violin, viola, cello, and double bass (e.g., Guettler, 1992; Winold and Thelen, 1994; Baader et al., 2005; Kazennikov and Wiesendanger, 2009; Schoonderwaldt and Demoucron, 2009; Kelleher et al., 2013; Verrel et al., 2013), wind instruments (e.g., Bejjani and Halpern, 1989; Cossette et al., 2008; Palmer et al., 2009; Albrecht et al., 2014), and percussion (e.g., Trappe et al., 1998; Dahl, 2004, 2018; Fujii et al., 2009a,b; Chen et al., 2016).

A general conclusion from this body of work is that movement patterns differ considerably, not only between players of different instruments, but also between players of the same instrument (Winold and Thelen, 1994; Dahl, 2004). This is to be expected considering the anatomical differences (Ortmann, 1962) and the many possible ways in which the motor system can achieve a particular end goal (e.g., pressing a piano key, Furuya and Altenmüller, 2013). Despite intrinsic differences in movement patterns, skilled performers generally appear to display:

- Timing stability and accuracy (Drake and Palmer, 2000; Fujii et al., 2011; Goebl and Palmer, 2013)
- Inter-joint coordination (Winold and Thelen, 1994; Furuya et al., 2011)
- Reduced limb load by proximal to distal movement organization. (Furuya and Kinoshita, 2007, 2008; Furuya et al., 2011; Goebl and Palmer, 2013; Verrel et al., 2014; Eriksen et al., 2018).
- Faster (and more brief) recruitment of motor units Lai et al., 2008; Fujii et al., 2009a,b).

Knowledge on biomechanical functions (as those listed above) can be objectively characterized and used to inform music performance pedagogy. In an effort to do this, Visentin et al. (2008) recorded joint angles and muscle activity for violinists' legato bowing at different tempi, and outlined different motor control phases displaying (1) increased bow acceleration and exertion; (2) constant bow acceleration and constant workload; and (3) approach of the physiological limits. After examining load patterns in joints and muscles, Visentin et al. (2008) concluded that both load quality and duration are important factors, and call for more research on how practice time can be optimized without inducing injury.

One way to assist and motivate a learner during practice time is through technology enhanced learning applications. In a survey on computer assisted musical instrumental tutoring (Percival et al., 2007) note the importance of making the students' individual practice time more efficient by helping the students with the task of self-analysis. A considerable number instrument tutoring systems have appeared during the past decades with approaches such as gamification (e.g., Klapuri et al., 2017) as well as augmented instruments (Pardue, 2017). Typically systems have a score based approach and often focus on only one type of instrument. The Imutus system (Schoonderwaldt et al., 2004) aimed at providing new learners of the recorder with lessons and automatic feedback on basic performance skills based on the audio signal. The framework was extended in its follow-up Vemus (Tambouratzis et al., 2008) to include more wind instruments and support for collaborative learning and group practice. These systems perform audio analysis and give feedback on whether the learner is producing the right note, tempo, phrasing and similar. Other systems extend this by providing feedback on the movements that produce the notes, such as fingering in violin playing (Dalmazzo and Ramirez, 2017) or the hand used in drumming (Kanke et al., 2017). In addition to the score based exercises, some systems employ haptic actuators to indicate which limbs or fingers the student should move (Holland et al., 2010; Lee and Choi, 2014), even providing “passive learning” of movement sequences (Huang et al., 2008).

Notably, most systems still largely focus on the score and the associated errors, with feedback related to muscle activation (Montes et al., 1993) or posture (Mora et al., 2006) being more rare. The overall focus on correctness of notes rather than overall musicianship and expression has been criticized by Xiao and Ishii (2016). Laying out a different kind of framework, (Xiao and Ishii, 2016) argue that rather than the focus on the basic technical movements (such as fingering or bowing) and the quantitative representations, system developers should emphasize the qualitative and first person perspective in their design. MirrorFuge (Xiao and Ishii, 2011) is an example of such an embodied approach, where users imitate video recordings of skilled piano performers projected on the piano. While MirrorFuge does not record or give feedback on users' movements, the system taps into the action-perception loop of the learner through imitation and copying of highly coordinated movements.

Among the systems and projects that target sound producing movements many have been focusing on violin playing (e.g., Yin et al., 2005; Ng et al., 2007; Schoonderwaldt and Wanderley, 2007; Ng and Nesi, 2008; Van Der Linden et al., 2011; Ramirez et al., 2018). The Digital ViolinTutor system (Yin et al., 2005) aimed at supporting the learner in the home practice situation through a combination of audio, instructor video and 3D animation of the playing movements. The system animates sequences from the score and the user can select sections of notes and parts of the movements (such as fingering on the board or bowing) to study more closely. Without input of the learner's movements, the feedback is based on pitch and timing of the notes produced. More extensive feedback on violin bowing movement is found in i-Maestro project (Ng and Nesi, 2008), and its implementation of an “Augmented mirror” (Ng et al., 2007). Using motion capture of the player, violin and bow, the augmented mirror shows the player with added visualizations of bowing trajectories, angles and where the bow is in contact with the string. A similar approach, also including measurement and visualization of bowing force, is used in the Hodgson plot (Schoonderwaldt and Wanderley, 2007). The Music Jacket (Van Der Linden et al., 2009, 2011) also captures the players' bowing movements using motion capture of the upper body, but instead of visualization, it provides the player with vibrotactile feedback to guide into proper violin position and development of bowing arm coordination. User tests showed some promise, although the effect was only persistent for some of the players post-tests. More recently, the Telmi project (Dalmazzo and Ramirez, 2017; Ramirez et al., 2018; Volta et al., 2018) aims to integrate both kinematic features as well as higher level features including sway and postural tension.

From the work reviewed, we note that systems that include movement instruction and feedback tend to concentrate on one instrument. Such an approach is practical for development purposes, considering the very different movement patterns between instruments and players. However, a more generic approach that could be used for multiple instruments would be an attractive solution for teachers and students alike, as this could offer a more comprehensive understanding of bodily effort and its relation to musical features.

As a general point, Percival et al. (2007) argue that targeting resources into helping the students with the task of self-analysis, i.e., helping students analyze what works and what needs improving, is likely to pay off better. Percival et al. (2007) suggest to focus on technical exercises and state that music students find it hard to judge their own performance. In this context, technology enhanced self-testing tools can be useful, however, real-time feedback to the learners might not be necessary. Rather, several authors (Percival et al., 2007; Van Der Linden et al., 2011) caution against distracting the students during playing. In view of the research reviewed one might ask what type of feedback technology enhanced learning tools should provide that will help a student to develop healthy and sustainable movement patterns. We propose movement fluency as a basis for such feedback.

## 3. Measuring Fluency

As a qualitative feature of movement, the characteristics of fluency are perhaps easier to judge by observers than calculated from measured data. Van Dokkum et al. (2012) defined fluency as gracefulness and ease of movement and assessed this for post-stroke patients by observers rating movement fluency. Whiting et al. (1987) referred to fluency as a qualitative parameter, but chose to measure it as the cross-correlation between a generated “ideal” velocity curve and the actual produced. While not specifically mentioning fluency, studies on music performance have used advanced musicians as “ground truth” when trying to characterize the kinematic features of skillful music playing. For instance, Chen et al. (2016) used qualitative ratings of percussion students' playing movements (including muscular relaxation) and resultant sound to evaluate a k-Nearest Neighbor classification of different timpani strokes. Volta et al. (2018) found a strong positive correlation between an aggregate measure of shoulder dynamics (including kinetic energy of shoulder and the first derivative of shoulder and elbow angles) and perceived violin playing skills as rated by expert violin teachers.

Most commonly, studies have attempted to characterize movement skill by estimating smoothness, as well as minimal effort and jerkiness (Nelson, 1983; Whiting et al., 1987; Hogan and Sternad, 2009). Rasamimanana and Bevilacqua (2008) applied the models for economical movement suggested by Nelson (1983), and correlated measured velocity and acceleration profiles during violin bowing with models of minimum cost. These cost functions either minimized acceleration transients (discrete and cyclical minimum jerk) or the area under the curve (minimum impulse). For repeated dtach strokes in an accelerando/decelerando task, they found a trapezoidal model best fitting the slowest strokes while the best fit for the fastest strokes was minimum jerk models. Rasamimanana and Bevilacqua (2008) concluded that the slow and faster parts of these tasks are best described by different optimization: while the slowest movements tend to optimize velocity variation, the faster ones optimized acceleration smoothness.

In a different approach, Kerr et al. (2013) proposed to deconstruct fluency as consisting of three parts: hesitation, coordination, and smoothness. While movement hesitation appears to be less relevant to our case of feedback during musical performance, we propose to quantify fluency as a combination of coordination and smoothness. Coordination related to movement optimization can contain several parts. As discussed above, the generation of highly skilled movements such as those in music performance, involves complex joint and muscle control by the central nervous system (Sosnik et al., 2004; Fujii et al., 2009b; Sakaguchi et al., 2014; Furuya et al., 2015; d'Avella, 2016). More specifically, these control challenges have been shown to depend on the ability to anticipate, segment, and *coarticulate* motor elements, all within the biomechanical constraints of the human body.

- *Coarticulation* can be generally defined as the integration and fusion of otherwise separate and distinct sequential movement elements into single units (Sosnik et al., 2004; Klein Breteler et al., 2006; Winges et al., 2013; Godøy et al., 2017). As movement skill and task refinement increases, sequences of individual motor elements are shaped toward a common “end goal”, with the ending of each element fitting with the start of the following one (Grafton and Hamilton, 2007). The amount of coarticulation is largely governed by the duration and rate of movement events. The fusion of events in coarticulation is also determined by anticipation of subsequent movement events, i.e., due to preparatory motion for upcoming events. For instance, violin players have been shown to adjust their fingering depending on whether a finger controls note onsets or pitch (Baader et al., 2005), and drummers can start the upward wrist movement in preparation for a louder stroke already during the preceding stroke (Dahl, 2004).
- *Proximal to distal movement organization*. A number of studies have rendered evidence on the fundamental kinematics of skilled music performance, showing that experienced musicians reduce limb load by making use of proximal joint motion, while novice performers tend to employ distal joint motion (Furuya et al., 2011; Goebl and Palmer, 2013; Verrel et al., 2014). Providing additional evidence of such patterns, Verrel et al. (2013) showed that novice cello players make use of proximal joint movement, while advanced performers tend to exploit distal joints during bow reversal exercises. In another field of musical performance, Furuya et al. (2011), investigated interjoint coordination in pianists through upper limb kinematics and muscular activity patters. Expert pianists were shown to have smaller movement range from distal joints and greater muscular activity from extrinsic upper limb muscles when compared against novice pianists. With increasing tempo, expert pianists were found to consistently make use of proximal rather than distal joints.

Until now, the underlying features of coarticulation of neuromuscular control and kinematics in music performance have primarily been studied independently and for single instruments. By combining these different components of fluency, assessment of movement skill can be made more standardized. One such general element is *smoothness*. Although perceptually very salient and linked to both motor skill acquisition and degeneration, finding a measure of movement smoothness that enables comparisons between subjects and tasks, is not straightforward (Rohrer et al., 2002; Hogan and Sternad, 2009; Djioua and Plamondon, 2010; Balasubramanian et al., 2012). Most commonly, measures of smoothness use the third derivative of position, “jerk” (e.g., Nelson, 1983; Hogan and Sternad, 2009; Larboulette and Gibet, 2015). A jerky motion would display a jagged velocity profile corresponding to less smooth movement. However, one concern with jerk-based measures is that calculated jerk vary depending on movement duration and how many sub-movements the motion consists of, making comparisons between different movers and studies difficult. Hogan and Sternad (2009) compared different jerk-based measures of movement smoothness with respect to their dependence on movement duration and components, finding evidence of higher reliability of dimensionless jerk-based measures. More recently, Balasubramanian et al. (2012, 2015) concluded that among jerk-based measures, only dimensionless jerk and the log dimensionless jerk are robust enough to be useful. Balasubramanian et al. (2012, 2015) further compared these jerk-based measures of smoothness to a measure based on the *Spectral Arc Length* (SAL, see Supplementary Material) of the movement velocity profile. The benefit of computing smoothness from the frequency spectrum of the velocity profile are reduced sensitivity issues derived from variability in movement amplitude and duration (Balasubramanian et al., 2012). However, SAL is not able to overcome issues related to number of movement components. Repetitive and rhythmic movements (such as bowing or drumming) by nature contain several movement components and (Balasubramanian et al., 2015) therefore conclude that smoothness measures cannot be applied to a rhythmic movement in its entirety, but suggest to calculate an aggregated smoothness across the individual movement components.

### 3.1. Movement Components

For the purpose of this study, it is useful to define the main movement categories that can be identified in a musician movement repertoire, and preferably also see them as generic, i.e., as applicable to performances on different instruments and in different types of music. We shall here present the main categories of impulsive, sustained, and iterative movements (see Godøy, 2006 for the sources of these terms), and also indicate how these are linked together:

- The basic feature of *impulsive movements* is that they are discontinuous, consisting of a short burst of effort followed by relaxation, such as in hitting, kicking, or rapid stroking. Sometimes referred to as “ballistic” motion in contexts of sports and everyday movement, impulsive movements are very common in music. In particular, percussion instrument performance display impulsive movements, but they can also be used for keyboard (c.f. Goebl et al., 2014) and string instruments, in the latter case with abrupt and fast motion such as with accented “martellato” bowing (Rasamimanana and Bevilacqua, 2008). Impulsive movements typically result in what we call impulsive sounds, such as that of percussion instruments and of the piano. Impulsive sounds typically have abrupt onsets immediately followed by decays, with the length of the decays dependent on the reverberant features of the instrument (Godøy et al., 2017).
- *Sustained movements* require a more or less continuous transfer of energy from the human body to the instrument (or to the human vocal apparatus), such as in continuous bowing, blowing, whistling or singing. Sustained movements result in what we refer to as sustained sounds, meaning sounds that are relatively stable in their overall loudness, pitch, and timbre, although there may be more minute variations in the sounds, such as by vibrato or in timbral fluctuations within the overall stationary appearance of the sounds (Nymoen et al., 2013; Godøy et al., 2017).
- *Iterative movements* consist of rapid alternations between effectors, such as in trills, or rapid back-and-forth movements of effectors such as in tremolos. Iterative movements are distinct from the two other categories in that they are neither sustained nor singular impulsive, but rather continuously fluctuating. Iterative movements typically use wrist tilting or wrist shaking, enabling very rapid motion with a minimum of effort, hence quite distinct from the other categories also in terms of effort and motor control (see e.g., Rasamimanana and Bevilacqua, 2008; Fujii et al., 2009a,b; Schoonderwaldt and Altenmüller, 2014). The sound output of iterative movements is very common in music, such as in drumrolls and other percussion textures, as well as in various ornaments on string and keyboard instruments (Godøy et al., 2017).

Changes in duration and rate of motion during sound-producing actions lead to interactions between these three motion categories, as well as to *phase transition events* (Haken et al., 1985). For instance, we may see a phase transition from impulsive to iterative movements if the movement event rate is increased, and conversely, a phase transition from iterative to impulsive movements if the motion rate is decreased (see e.g., Rasamimanana and Bevilacqua, 2008). Similarly, we may see a phase transition from sustained to impulsive movement if the duration of the sustained movement is shortened beyond a certain threshold. The importance of phase transition events for fluency in music performance lies in the need to fuse otherwise discontinuous movements into superordinate and smooth movement chunks, or conversely, the splitting up of continuous and smooth movement chunks into smaller and more discontinuous movements entities. Phase transition events are also useful to highlight stability in bimanual movements. Fujii et al. (2010) investigated stability of movement phase between hands from drummers and non-drummers during rapid bimanual drumming. Not surprisingly, the drummers showed greater stability in their bimanual coordination compared to non-drummers.

For our purpose of characterizing fluency in music performance, we categorize movements in music performance as impulsive, sustained, and iterative, allowing the comparison of skill across different instrumental families. For instance, the sound producing movements used to excite the vibrating structures differ in cello and drum playing (sustained vs. impulsive). However, while these players display different kinds of movement repertoire, iterative movements are frequent in both drumming and cello bowing.

A useful technology assisted learning system for music performance also needs to be able to assess fluency across various levels of skill. One way would be to assess how movement control differs for different playing tempi. If we imagine a space of tempi and dynamic range, each player has a “playability area” where playing is manageable (c.f. Dahl, 2006; Visentin et al., 2008). For novices, the area is fairly small (slow tempo, medium to loud sound level) wheareas advanced performers are able to stretch it to include also faster tempi. However, also experts will have difficulties to maintain stable performance for extreme combinations of tempo and dynamic level (Dahl et al., 2011).

To summarize, increasing and decreasing movement tempi for basic movements in drumming and bowing are likely to display differences in movement fluency (and organization) between trained and untrained players while providing simple tasks that also new students are able to do. To investigate such manifestations of fluency, we recorded and analyzed movements of cello players and drummers of varied expertise playing at gradually accelerating and decelerating tempi. Furthermore, we quantifed the end-effector movement smoothness, and evaluated coordination from movement and muscle activity data using principal component analysis.

## 4. Materials and Methods

We recorded cello bowing and drumming movement data for players of varying skill. During the experiment we captured full body motion capture for a number of tasks. In this paper, however, we concentrate on motion from the right hand effectors in a transition task of accelerando followed by decelerando bow and drum strokes, respectively.

### 4.1. Participants and General Apparatus

One advanced and two apprentice drummers (3 male, all right-handed, age mean = 29.67 years, age *SD* = 10.97), and one advanced and two apprentice cello players (2 male, 1 female, all right-handed, age mean = 20.67 years, age *SD* = 13.61) took part in the study. Advanced players had at least 15 years of instrument experience and were teaching their instrument professionally, while apprentices were students at a music academy. All participants gave their written informed consent prior to the experiment and answered a brief questionnaire about musical training experience, age, handedness as well as an online survey giving their Ollen Musical Sophistication Index (OMSI,^1^ Ollen, 2006), shown in Table 1. The OMSI score is a number between 0 and 1,000 indicating the probability (percent × 10) that a respondent would be classified as “more musically sophisticated” by a music expert. Participants' years of instrumental practice and OMSI scores are shown in Table 1. Participants were informed that their participation was voluntary and that they could withdraw from the study at any point in time. The study obtained ethical approval from the Norwegian Center for Research Data (NSD), with project number 59876.

**Table 1:** Instrumental practice and Ollen Musical Sophistication Index (Ollen, 2006) for players.

**Participant**	**Instrument**	**Years of instrument practice**	**OMSI score**
D1	Drums	24	755
D2	Drums	7	58
D3	Drums	10	153
C1	Cello	16	984
C2	Cello	10	124
C3	Cello	2	46

Recordings took place in a motion capture lab at Department of Musicology, University of Oslo. We recorded participants movements using a twelve-camera optical motion capture system (Qualisys Oqus, Sweden) at a frame rate of 400 Hz, tracking the three-dimensional positions of reflective markers attached to each participant's body and instrument. For this paper, however, we focused on smoothness of trajectories of end-effectors from the markers on the cellists' right hand and the drummers' drumstick in our analysis. Future studies will look into body segments, posture, and angular characteristics of movement from the data gathered.

Muscle activity was recorded at 2,000 Hz as surface EMG using Delsys Trigno (Boston, MA). An analog trigger unit was used to synchronize both motion capture and EMG acquisitions in time. Details on the electrode placement for each instrument groups is given in the following sections while we present details on the data processing in Supplementary Materials.

### 4.2. Drums Setup and Task

**Figure 1:**
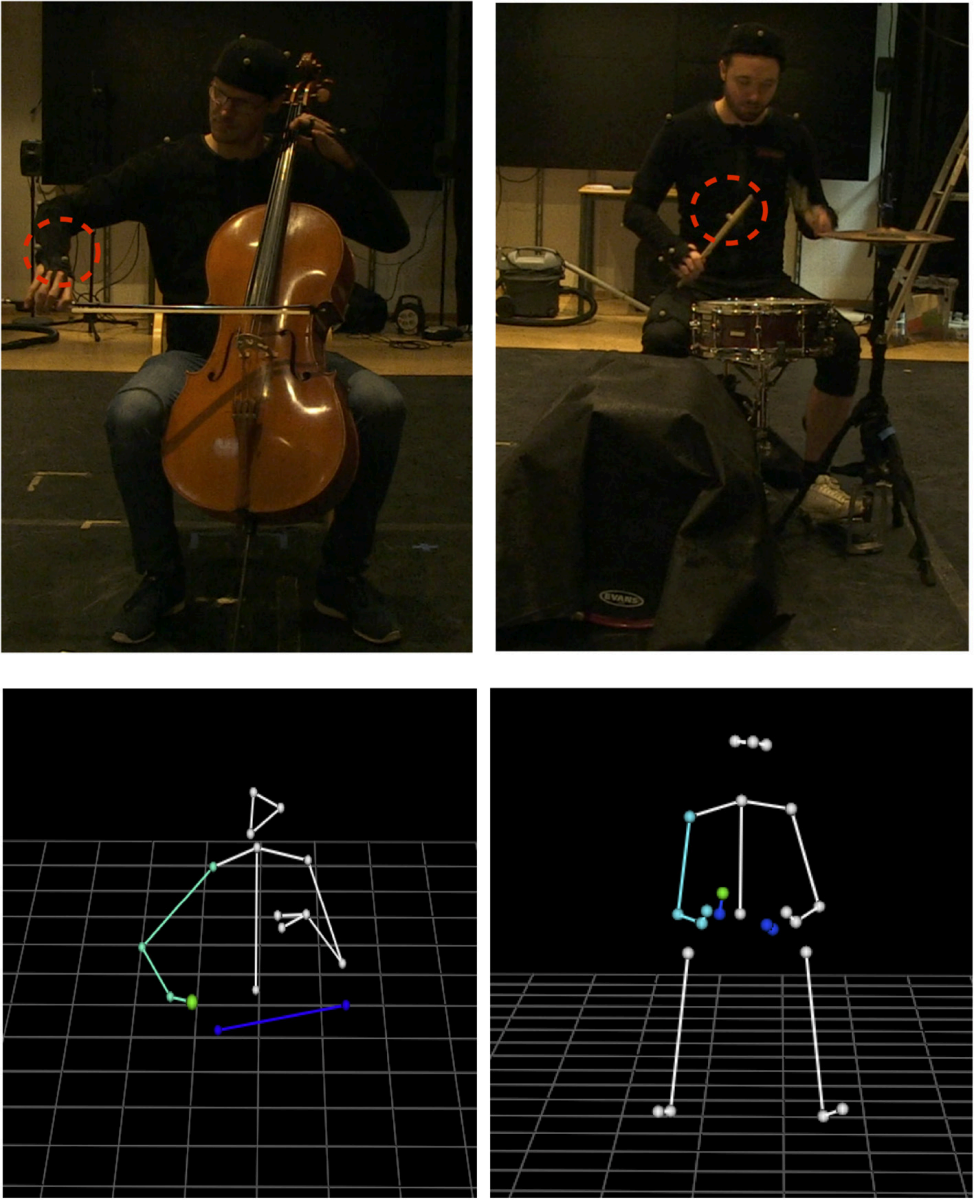
Experimental setup and 3D reconstruction of markers from motion capture data for the cello **(Left)** and drums **(Right)** tasks, with the right arm 3D reconstruction shown in green and blue, respectively. The bass drum is covered with black cloth to minimize reflections interfering with the tracking of optical markers. Note the placement of the markers on the drum sticks which is some distance away from the tip so as not to interfere with playing. Written informed consent was obtained from participants for publication of images.

The drumming motion data was recorded in a pre-defined capture volume including and surrounding the drum kit and in the laboratory global coordinate system defined through calibration. The drummers were seated on a drum throne and played a basic drum kit consisting of a hi-hat, a snare drum, and a bass drum as shown in Figure 1. The drummers could adjust the height of the drum throne as well as the stands to the appropriate height. Passive markers were located in the following anatomical landmarks as seen in Figure 1: 1, front head; 2, right back head; 3, left back head; 4, right shoulder; 5, left shoulder; 6, spine; 7, middle of the sacrum; 8, left elbow; 9, right elbow; 10, left ulna; 11, right ulna; 12, left hand-dorsal; 13, right hand-dorsal; 14, left knee; 15, right knee; 16, left heel; 17, right heel; 18, left foot-fifth toe; 19, right foot-fifth toe. Two markers were placed on each of the player drumsticks (located approximately at 1/4 and 3/4 of the length of the stick). Wireless EMG electrodes were placed on the location of the upper trapezius, triceps, forearm flexor and extensor, and gastrocnemius following suggestions from SENIAM ^2^.

**Figure 2:**
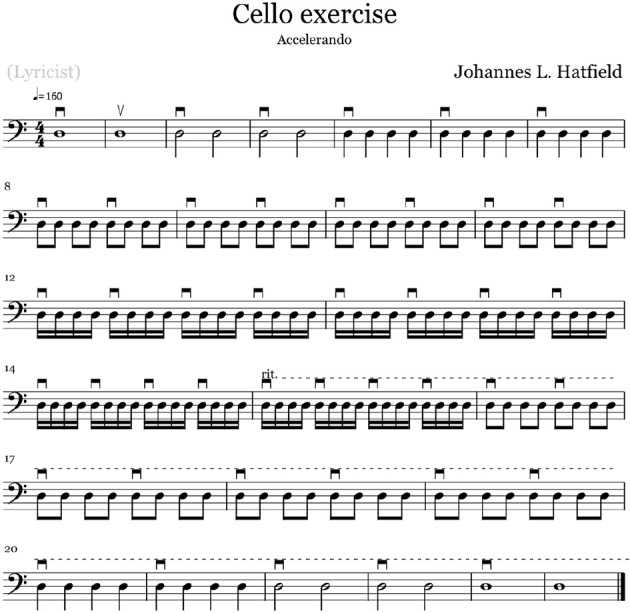
Score from the *Accelerando* cello exercise. Symbols above the notes denote up and down bows. For the drumming task the score would be the same but with the symbols exchanged for R and L, indicating right and left hand respectively.

The drumming task selected for analysis consisted of single strokes with alternating hands gradually accelerating in tempo, producing a single stroke roll. Figure 2 shows an example score of the task. Players started at a slow, comfortable tempo and gradually increased tempo until reaching maximum speed, after which they gradually decelerated to a comfortable slow tempo.

### 4.3. Cello Setup and Task

The recording of cello players' movements was similar to that of drummers. The calibrated capture volume included and surrounded the cello player and the instrument. The system recorded the positions of 15 passive markers placed on player and bow, of which we selected the right hand marker for analysis. 15 passive markers were located at: 1, front head; 2, right back head; 3, left back head; 4, right shoulder; 5, left shoulder; 6, spine (T1); 7, middle of the sacrum; 8, left elbow; 9, right elbow; 10, left ulna; 11, right ulna; 12, right hand-dorsal; 13, left index finger middle phalanx; 14, left ring finger middle phalanx. The implemented marker set and the resulting 3D model can be seen in Figure 1.

Surface EMG electrodes were placed on the location of the middle deltoid, upper trapezius, triceps, and forearm flexor and extensor, again following suggestions from SENIAM for skin preparation and optimal placement identification.

Similarly to the transition task for the drummers, the cello players were asked to perform right-hand plain sixteen notes, starting at a comfortable tempo and gradually accelerate until maximum tempo and then decelerate. A transcript of the score used to instruct participants can be seen in Figure 2.

## 5. Analysis

Our analysis focused on smoothness calculation from the velocity of the right effector in combination with estimating coarticulation patterns as measured by principal component analysis of surface electromyography. While fluency and smoothness are likely to be visible anywhere in the movement chain, we made slightly different choices as to what part to use for the analysis depending on the instrument played. For the cello bowing we concentrated the analysis to the velocity of the marker on the hand, whereas the stick marker was chosen for the drumming. For the drumming movements, we instead selected the velocity of the drumstick marker. For percussion, the striking velocity has been related to the produced sound level and has been linked both to type of stoke played as well as expertise (Dahl, 2011; Chen et al., 2016). We here briefly explain the analysis made and refer to the Supplementary Material for more details on both kinematic and EMG analysis.

### 5.1. Kinematic Analysis

Three-dimensional position data from the cello and drumming trials was processed using the MoCap toolbox for Matlab (Burger and Toiviainen, 2013). We calculated the velocity profile (i.e., magnitude of the linear velocity) for both bowing and drum strokes, applying a second order Butterworth smoothing filter. We then segmented the strokes based on identified peaks in the velocity profile and calculated the *inter-onset interval* (ioi) between strokes. From the normalized Fourier magnitude spectrum of each individual stroke we then obtained the *spectral arc length (SAL)* (Balasubramanian et al., 2012, see also Supplementary Material). In essence, the smoother the bowing or drumming stroke, the more positive SAL. For the movements used in this paper SAL stayed negative throughout.

We defined the different phases during the accelerando-decelerando task based on the tempo expressed in ioi as: **slow** (>0.6 s); **transition** (0.4–0.6 s); **fast** (0.2–0.4 s); and **very fast** (< 0.2 s). Since we here only include right arm, the calculated iois for drummers include one stroke, while those for cellists includes both up- and down bows, halving iois. However, since the drummers were striking with both hands the temporal regions are comparable with respect to number of notes played. The series of calculated SAL metrics for the phases of each participant were then used as a basis to compare smoothness of players' movements.

### 5.2. EMG Analysis

Details on the data treatment of muscle activity signal from selected muscles on the dominant arm for each participant can be found in the Supplementary Material. In brief, we segmented the signal using the temporal locations identified from the segmentation of kinematic data and computed a single smoothed and normalized EMG signal for each participant, muscle, and identified stroke.

We then applied time-varying Principal Component Analysis (PCA) (see Supplementary Material and Santello et al., 2002; Klein Breteler et al., 2006; Winges et al., 2013) in order to evaluate coarticulation from burst patterns across strokes during the exercises. The PCA results in a number of principal components (PCs) which are ranked in terms of the amount of variance in the data explained by each component (see Santello et al., 2002). Each stroke can then be computed as its mean EMG amplitude plus the sum of the ranked PCs (with PC1 explaining the most variance, PC2 the second most variance etc.). For each participant and playing phase, we then get a set of PCs, each representing the combined muscle activation pattern during a stroke. Due to the limited number of strokes for some playing phases for the cellists, the activation patterns for cello players included both up- and down strokes. This means that muscle activation patterns were calculated across different phases of the movement which would weaken the outcome of the PCA, but it was a necessary choice to allow us to calculate the PCA for all phases. We set an arbitrary threshold of 60% of cumulative variance accounted and used this threshold in subsequent analysis, with the number of PCs needed to achieve the 60% used as input to a regression model explained in the results section.

## 6. Results

**Figure 3:**
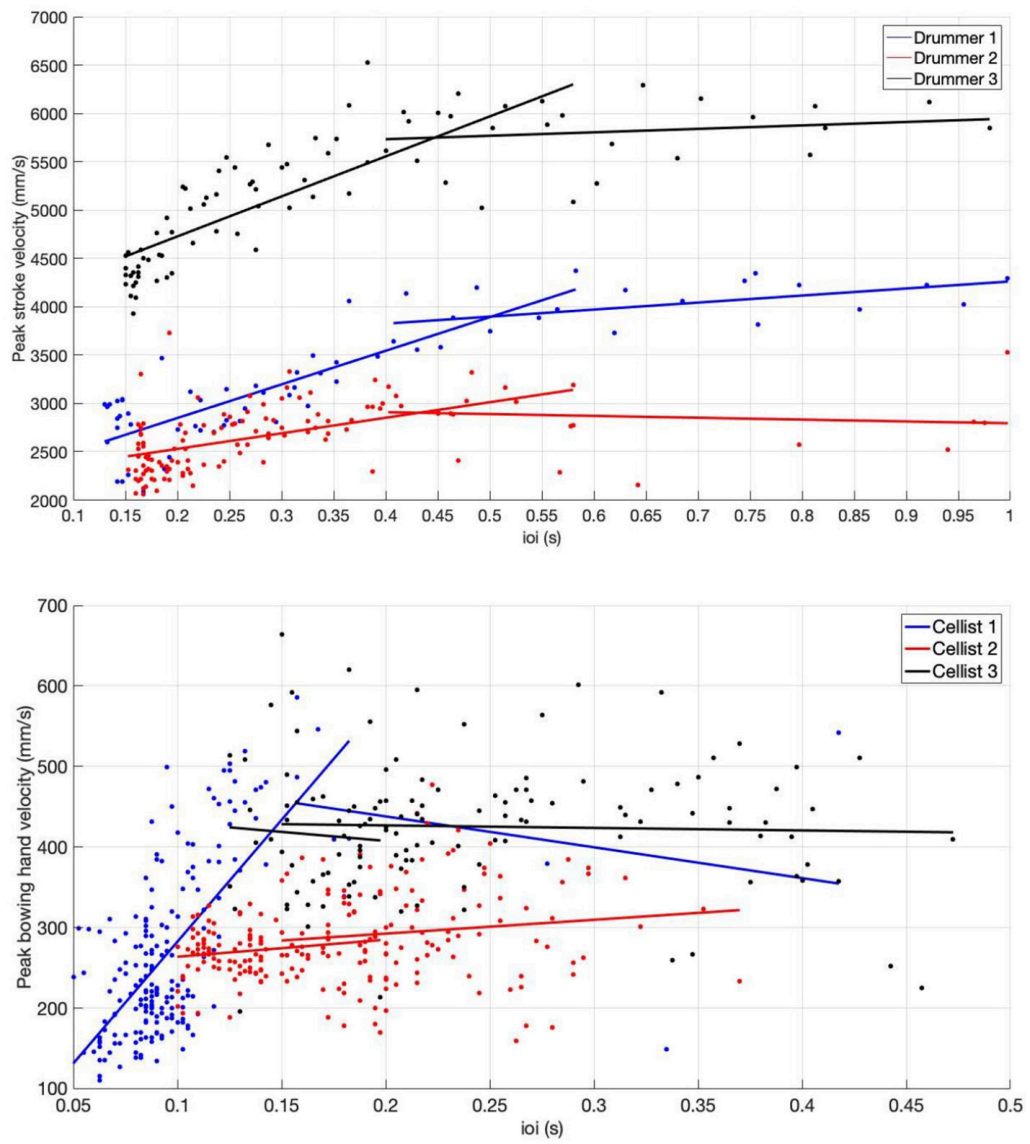
Measured peak velocity vs. inter onset intervals with fitted linear lines to illustrate the playability regions for the drummers **(Top)** and cellists **(Bottom)**. Note the smaller values in ioi for the cellists due to the separation of up- and down bows in ioi. For drummers D1 and D3 (top), the decrease in peak velocity when accelerating from slow to fast (going from right to left along the ioi-axis) is clearly seen. Player D1 is able to push the limit of “break down” further than the students. For the cellists the different phases are mainly seen for player C1 since students C2 and C3 had difficulty performing at faster tempi.

As an illustration of the playability area discussed at the end of section 3, we plotted the measured peak velocity of each individual bow and drum stroke during the acceleration-deceleration task against the measured inter-onset interval (ioi) between strokes. Figure 3 shows the produced peak velocities at different stroke duration for players of different skill. Similarly to Visentin et al. (2008) we also indicate the phases. Linear regression lines were fitted to velocity data for iois in “slow” and “fast” temporal regions, respectively. The lines were extended past their data regions to illustrate the transition where the regions intersect. In addition to the playability area for players, Figure 3 also reveal differences in how the players interpreted the instructions. Specifically, some players moved very quickly from one phase to another, which resulted in very few strokes in the slow phase for several players. Since a minimum number of strokes are needed to compute the input matrix for the PCA, we therefore opted to include both up- and down strokes in order to increase the number of data points. Although each point represents one movement, the actual onset between events differ since the drummers used two hands, producing events with double density. In the following we present the results for each instrument in more detail.

### 6.1. Drums

The top panel in Figure 3 shows the individual peak velocities vs. ioi for drummers D1-D3. As can be seen, the range of iois across the task is roughly 0.12–1 s, with gradually decreasing peak velocity for faster strokes. The decrease in peak velocity is expected, as there is little time to accelerate the stick during fast playing. Notably drummer D2, who was struggling with the exercises, display an overall low level in peak velocity also for longer iois.

**Figure 4:**
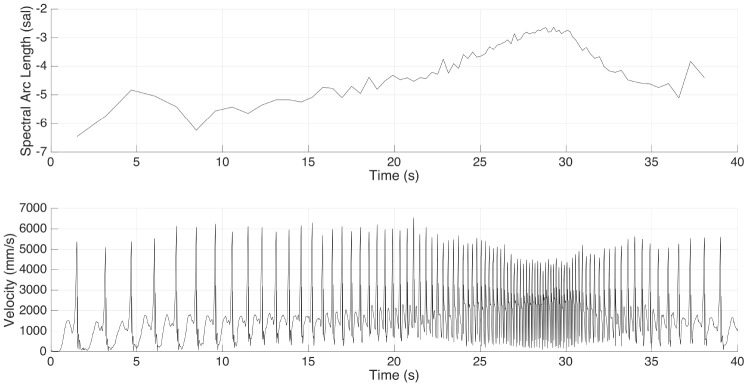
**Top**: Drum stick spectral arc length metric evolution from participant D3 during the single roll task. **Bottom**: Drum stick velocity profile from participant D3 during the single roll task.

An example of the gradual reduction of peak velocity for faster playing can be seen in the bottom panel of Figure 4. The figure displays measured velocity of the drums stick marker for participant D1 during the single roll task (bottom panel) with the computed SAL in the top panel. The drum stick velocity profile from participant D3 was consistent with the acceleration-deceleration nature of the task, with higher values for the slow tempo during for the first 10 s and clear decrease in peak velocity for the shorter iois. Throughout the exercise SAL varied with peak drum stick velocity from a minimum of −6.46 to a maximum of −2.63 (mean = −3.97, sd = ±0.96).

**Figure 5:**
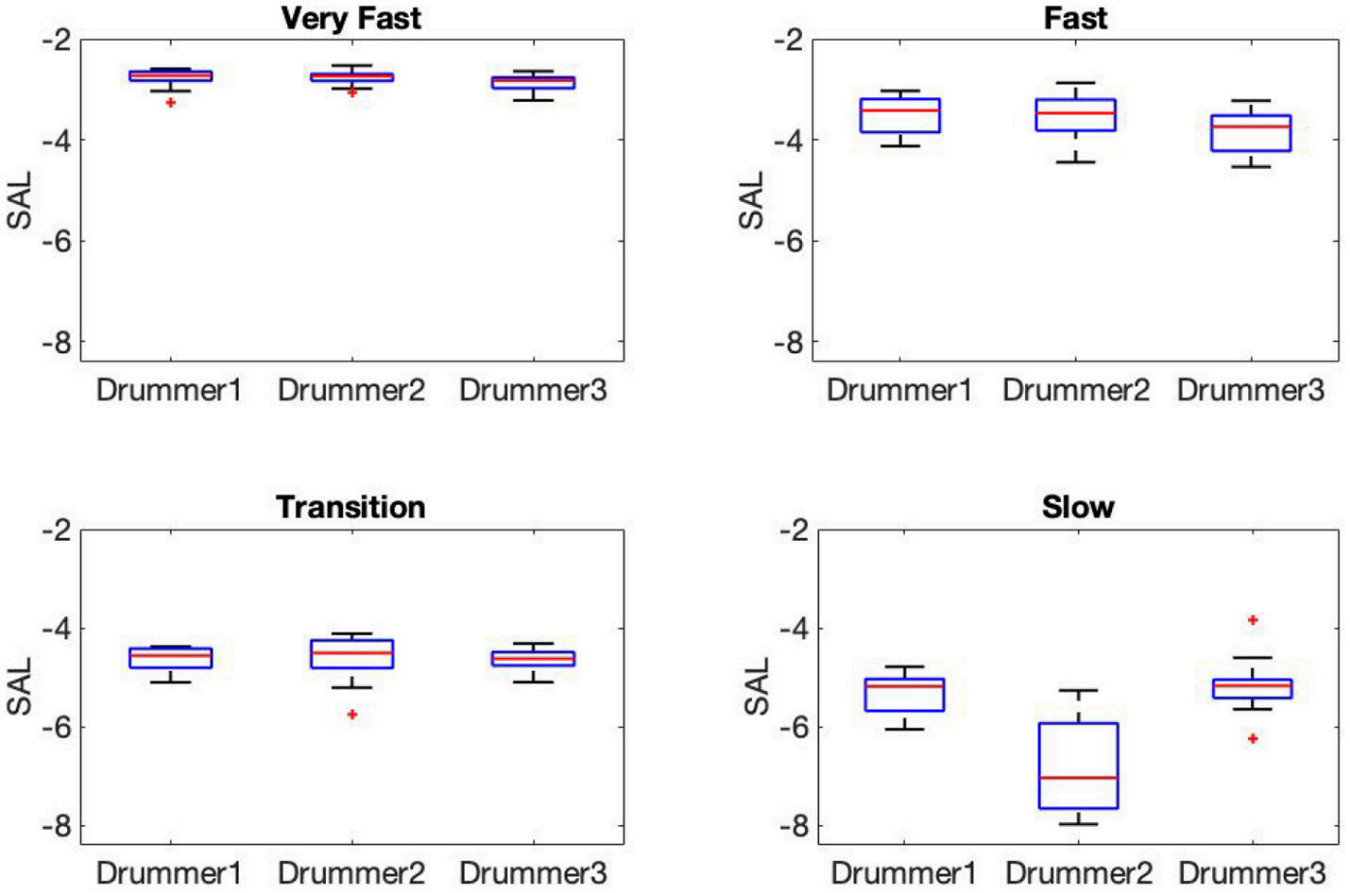
SAL metric for the three drummers computed across different phases: Very fast (top left panel), fast (top right panel), transition (bottom left panel), and slow (bottom right panel). Each box displays the range of upper and lower quartile values with the median as a red line in the middle. Whiskers are showing maximum and minimum values. As can be seen, SAL decreases with decreasing inter-onset intervals for all players but more so for D2. The lower SAL at slower tempi is explained by the sharper velodicty curve at impact for the more separated strokes (see Figure 4).

Figure 5 displays box plots showing median and ranges of SAL across drummers and phases. As can be seen, smoothness tended to increase at faster tempi (*medianSAL*_*D*1_ = −2.76, *medianSAL*_*D*2_ = −2.76, *medianSAL*_*D*3_ = −2.86 for the very fast phase; *medianSAL*_*D*1_ = −5.18, *medianSAL*_*D*2_ = −7.04, *medianSAL*_*D*3_ = −5.17 for the slow phase). One explanation for this is the general higher peak velocity produced for strokes at slower tempi (see Figure 3). Moreover, differences between participants were clearer during the fast and slow phases [Kruskal–Wallis χ^2^_(2)_ = 12.2, *p* = 0.002, and χ^2^_(2)_ = 10.21, *p* = 0.006, respectively], while smoothness during the transition phase was observed to be more consistent [χ^2^_(2)_ = 1.05, *p* = 0.592]. Higher peak velocity at impact results in a less smooth movement curve due to the abrupt changes of direction for the stick. With increase in tempo however, movement amplitude decreases and with less runway the striking velocity, and resulting sound level is reduced.

The contribution of each of the first 10 principal components and their cumulative variance for each playing phase can be seen in Figure 7. In most cases the first few PCs contribute with the main part but as expected there are differences across the players and tempi. In particular, the number of PCs needed to explain 60% of the variance in muscle activity increases for all players during the fast phase, but more evidently for player D2.

In Figure 9, the waveforms from the PCs are shown for each muscle and player during the fast phase. The waves are centered around the impact of the stroke (velocity peak) at 150 ms. For all players, the central region of PC1 was dominated by bursts of activity in the trapezius, with the later part of the component dominated by bursts from the triceps and lower activity from the wrist flexor. For participant D1, PC2 waveforms showed high frequency bursts from the three analyzed muscles, reaching maximum amplitude toward the last segment of the PC. Activity from participant D2 in PC2 was characterized by dominance of bursts from the triceps toward the middle section of the PC, while waveforms from D3 exhibited anticipated activity from trapezius followed by coinciding bursts from flexor and triceps.

The PCA waveforms from drummers D1-D3 in the transition phase are shown in Figure 10. Waveforms from PC1 showed consistent patterns across participants, with dominant burst from trapezius and triceps toward the second half of the stroke. Activity from participants D1 and D3 was characterized by initial bursts from triceps. PC2 from D1 and D3 showed high frequency bursts across the stroke, with amplitude maximum from the flexor toward the second half of the stroke. Participant D3 exhibited a series of trapezius bursts across the stroke and initial burst from flexor, with triceps activating in the final 100ms of the stroke.

The waveforms from PC1 and PC2 for the slow phase revealed inconsistent patterns across participants, as shown in Figure 11. For participant D1, PC1 was characterized by predominant sustained activity from the triceps preceding the peak of the stroke, while waveforms from participant D2 showed bursts from trapezius throughout the whole stroke duration and lower amplitude bursts from the triceps. In participant D3 U-shaped waveforms characterized activity from both trapezius and triceps. Waveforms from PC2 in participants D1 and D2 exhibited high frequency activity from all three muscles reaching maximum amplitude toward the final part of the stroke.

### 6.2. Cello

**Figure 6:**
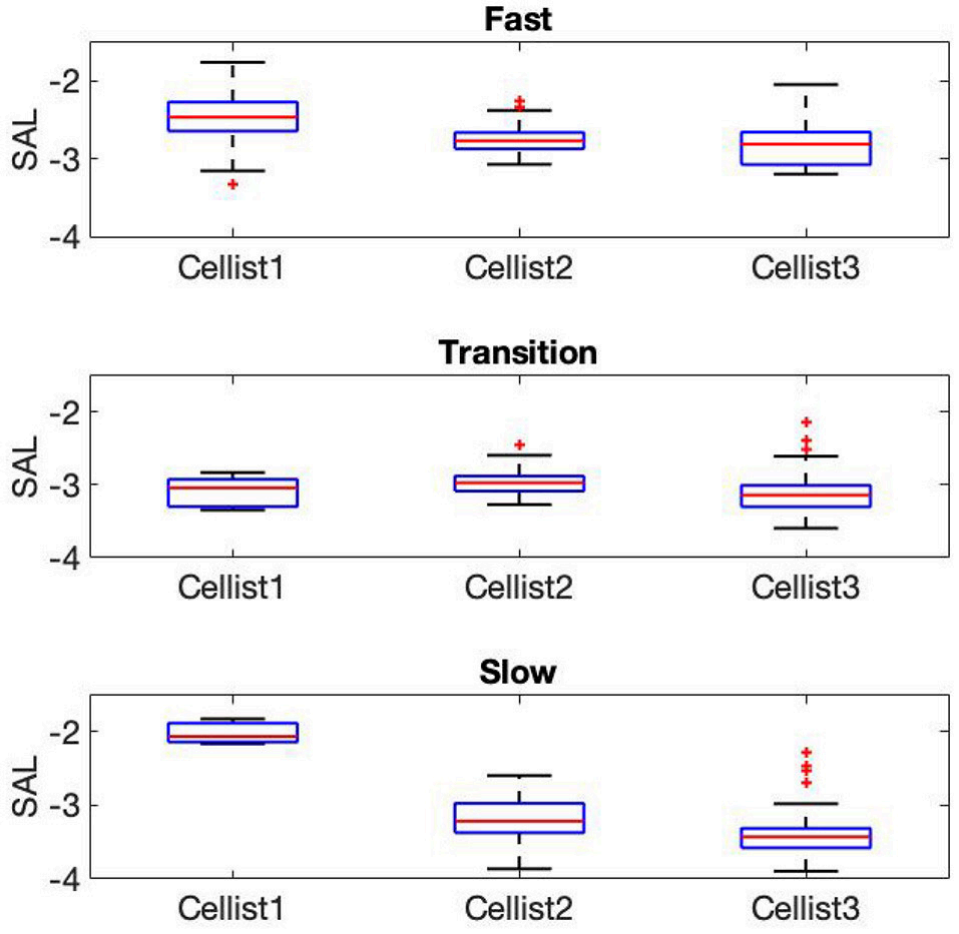
SAL metric for the three cellists computed across different phases: Fast **(Top)**, transition **(Middle)**, and slow **(Bottom)**. Each box displays the range of upper and lower quartile values with the median as a red line in the middle. Whiskers are showing maximum and minimum values.

The bottom panel in Figure 3 displays the measured peak velocity vs. ioi for the three cellists, C1-C3. The differences in variability and playability area between players is clearly seen. Note that the the bottom panel in Figure 3 includes both up- and down bows and consequently have iois in half the range compared to that of the drummers (top panel). Since only player C1 was able to achieve very fast tempo during the exercise, we restricted the analysis to the slow, transition, and fast phases for the cellists. Furthermore, as can be seen by the number of points within the slow phase, cellist C1 started the transition phase almost immediately, resulting in only three strokes qualifying for the slow phase. In order to make a comparison possible between cello and drumming participants across all phases we decided to include both up- and down bows. Median and ranges of SAL across cellists and phases are shown in Figure 6, with greater smoothness of bow movement at faster tempi for all participants [Kruskal–Wallis χ^2^_(2)_ = 64.5, *p* < 0.001, *medianSAL*_*C*1_ = −2.46, *medianSAL*_*C*2_ = −2.76, *medianSAL*_*C*3_ = −2.80]. The transition phase was characterized by smaller differences in SAL values across participants [χ^2^_(2)_ = 15.13, *p* < 0.001, *medianSAL*_*C*1_ = −3.04, *medianSAL*_*C*2_ = −2.97, *medianSAL*_*C*3_ = −3.14], while the slow phase displayed less smooth movements for participants C2 (*medianSAL* = −3.21) and C3 (*medianSAL* = −3.43) compared to participant C1 [*medianSAL* = −2.067, χ^2^_(2)_ = 32.25, *p* < 0.001].

**Figure 7:**
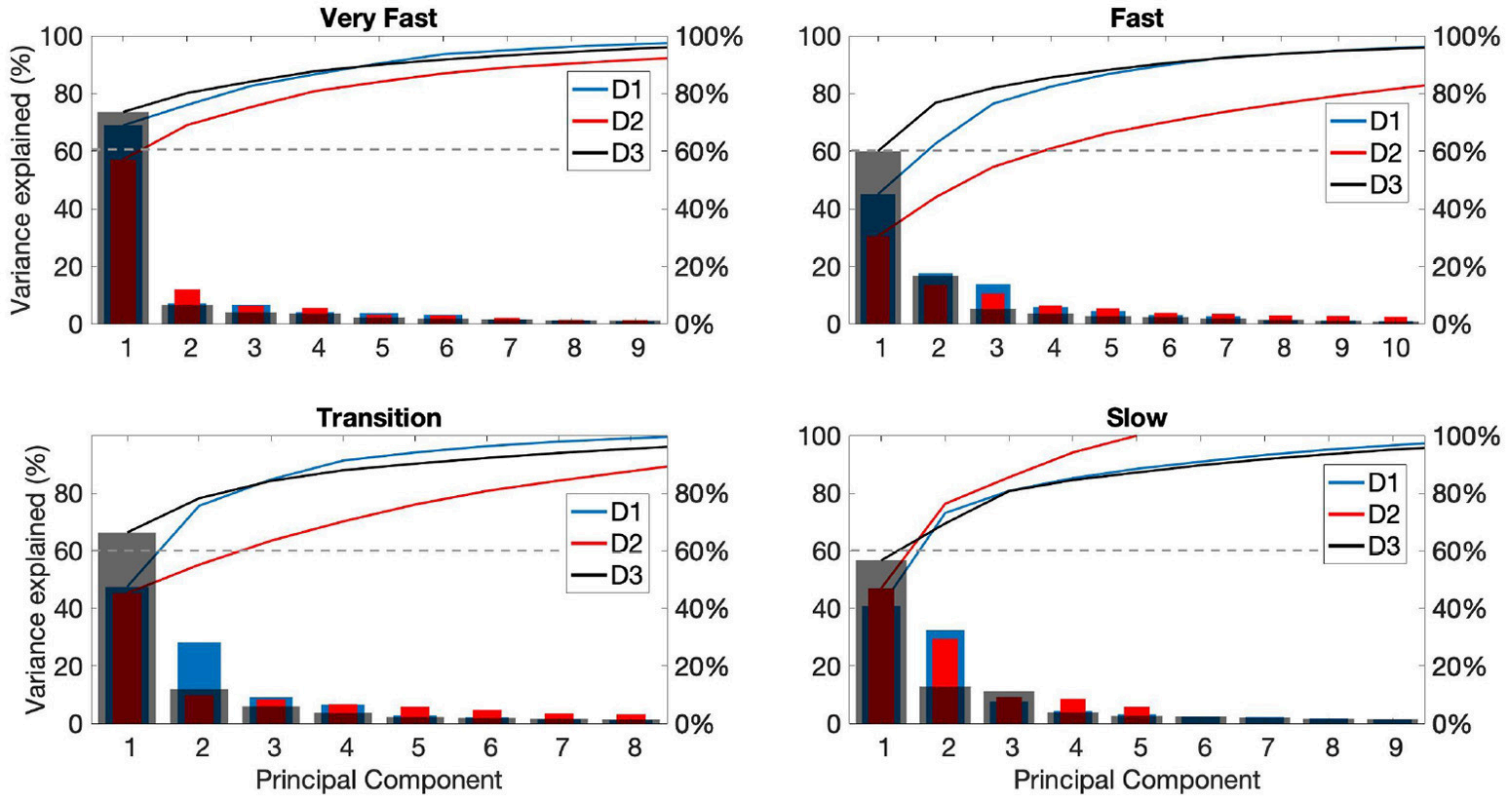
Principal component (PC) analysis results for drummers D1-3 for the different playing phases: fast (top panel), transition (middle panel), and slow (bottom panel). Each panel shows the percentage of variance accounted for by PCs 1-10 for players D1 (blue), D2 (red), and D3 (gray).

**Figure 8:**
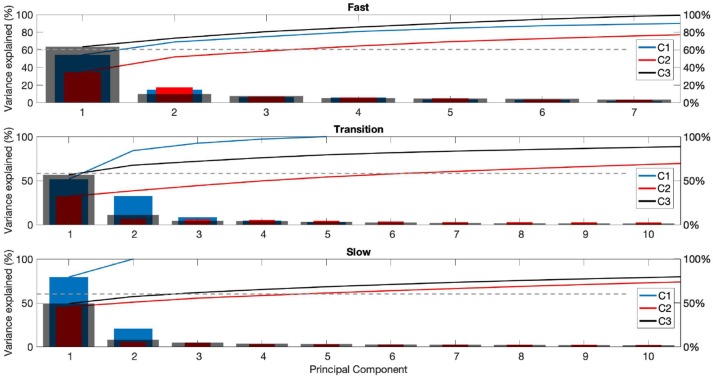
Principal component (PC) analysis results for participants C1-3 for the different playing phases: very fast (top left panel), fast (top right panel), transition (bottom left panel), and slow (bottom right panel). Each panel shows the percentage of variance accounted for by PCs 1-10 for players D1 (blue), D2 (red), and D3 (gray).

Results from the PCA for the cello exercises are shown in Figure 8, displaying the contribution of each of the first 10 principal components to bowing strokes for each playing phase for each participant (C1-C3). Participant C1 exhibited greater consistency across phases, with PC1 accounting for over 50%, and PC1 and PC2 cumulative variance exceeding 60% in all three phases. During the fast phase, the first two PCs accounted for over 60% of the variance in the EMG patterns across bow strokes for all participants.

**Figure 9:**
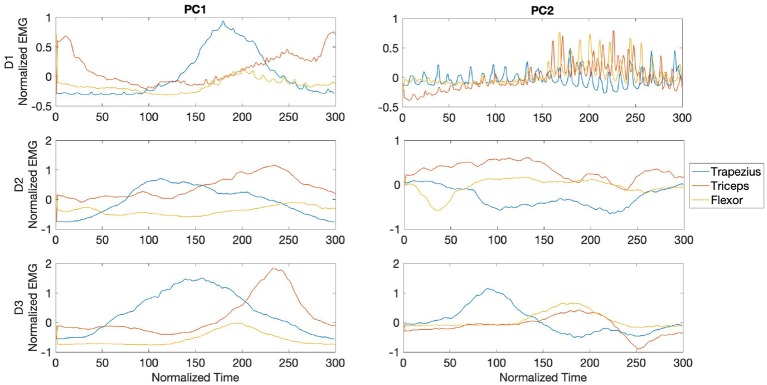
Waveforms showing the two principal components of muscle activation across the trapezius (blue), triceps (orange), and wrist flexor (yellow) muscles for drummers D1-3 in the fast phase (top right panel in Figure 7). Each panel shows the activation pattern centered around stroke onset (150 ms). The difference in activation patterns across players is clearly seen.

**Figure 10:**
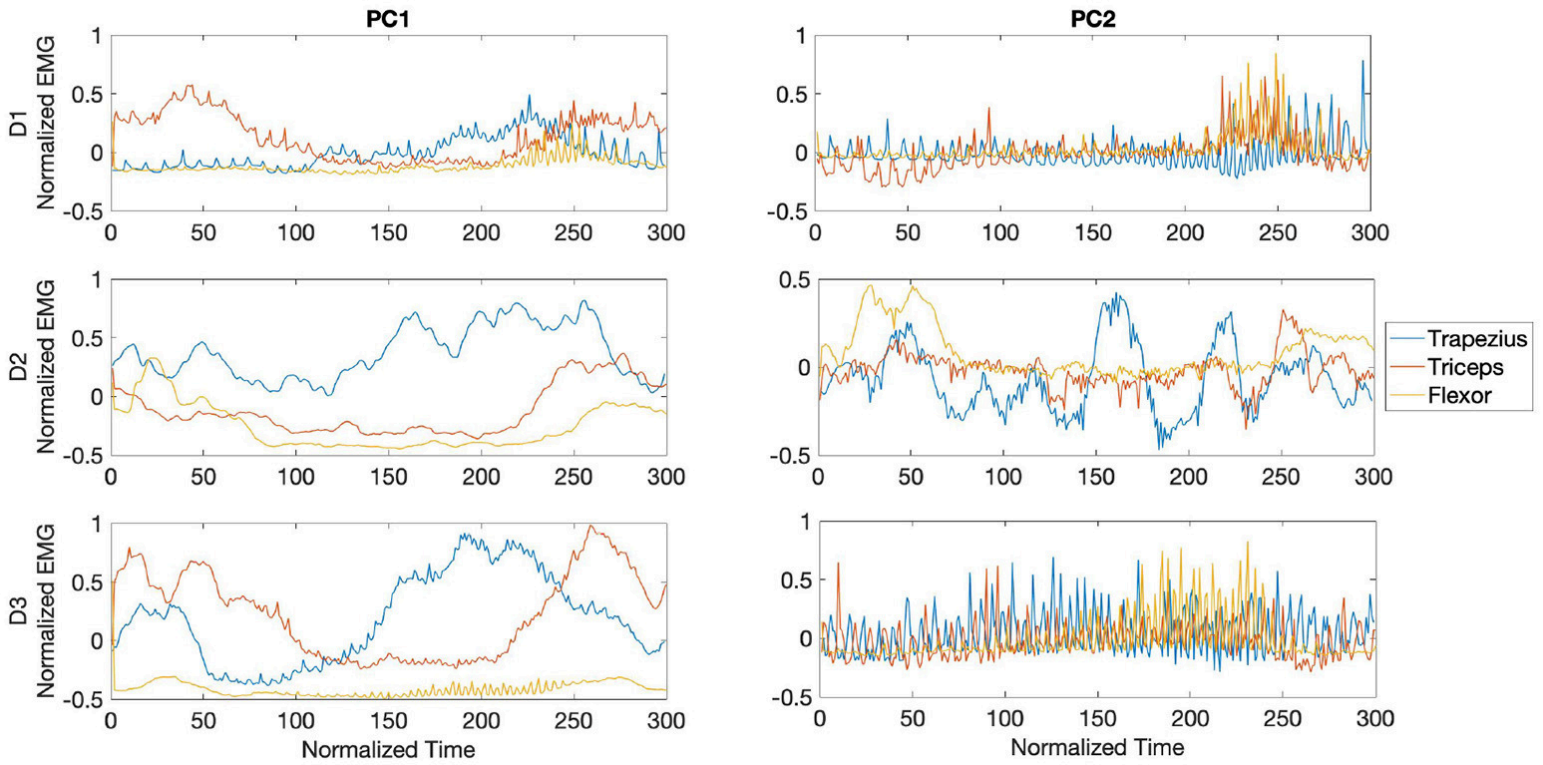
Waveforms showing the two principal components of muscle activation across the trapezius (blue), triceps (orange), and wrist flexor (yellow) muscles for drummers D1-3 in the transition phase (bottom left panel in Figure 7). Each panel shows the activation pattern centered around stroke onset (150 ms).

**Figure 11:**
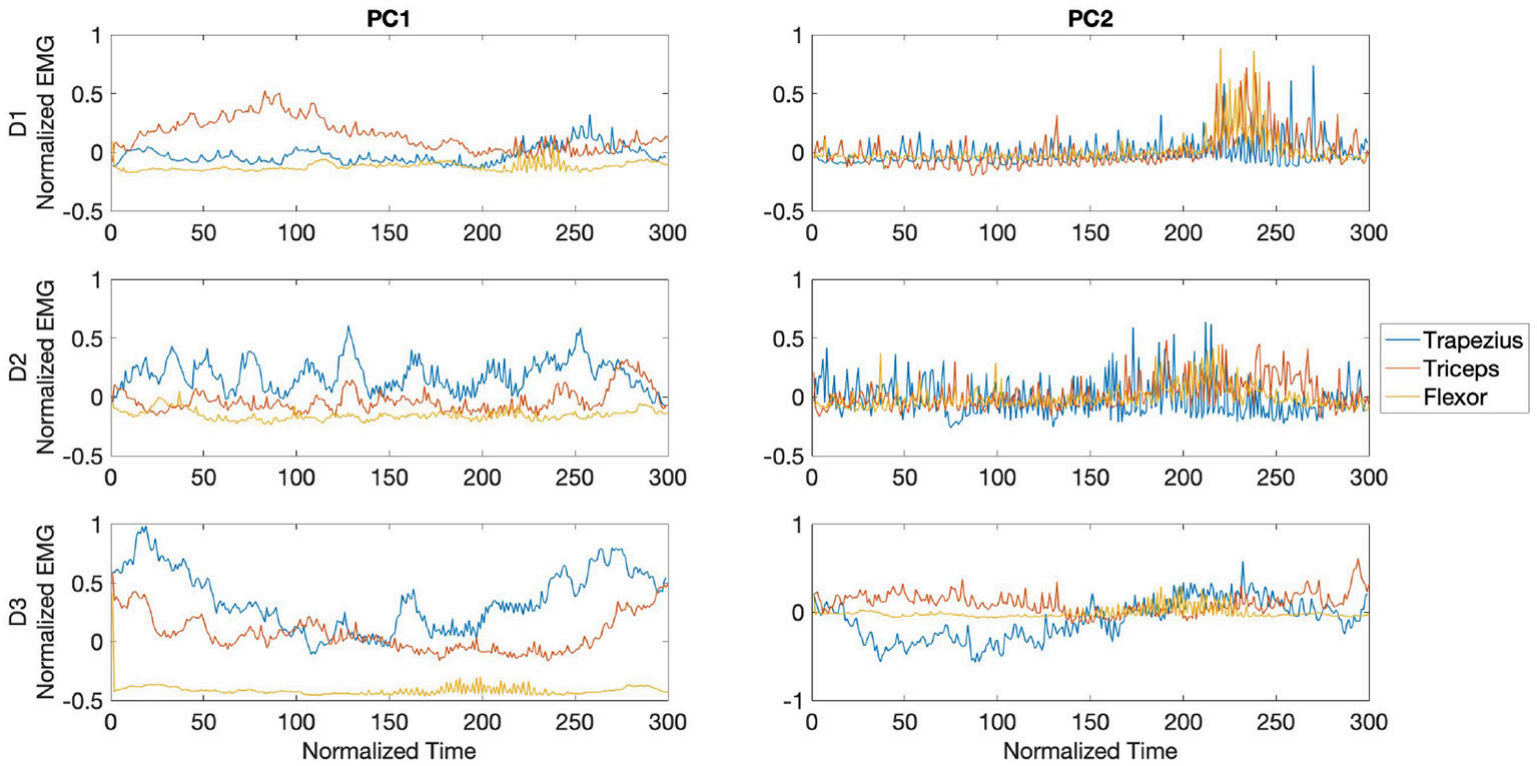
Waveforms showing the two principal components of muscle activation across the trapezius (blue), triceps (orange), and wrist flexor (yellow) muscles for drummers D1-3 in the slow phase (bottom right panel in Figure 7). Each panel shows the activation pattern centered around stroke onset (150 ms).

**Figure 12:**
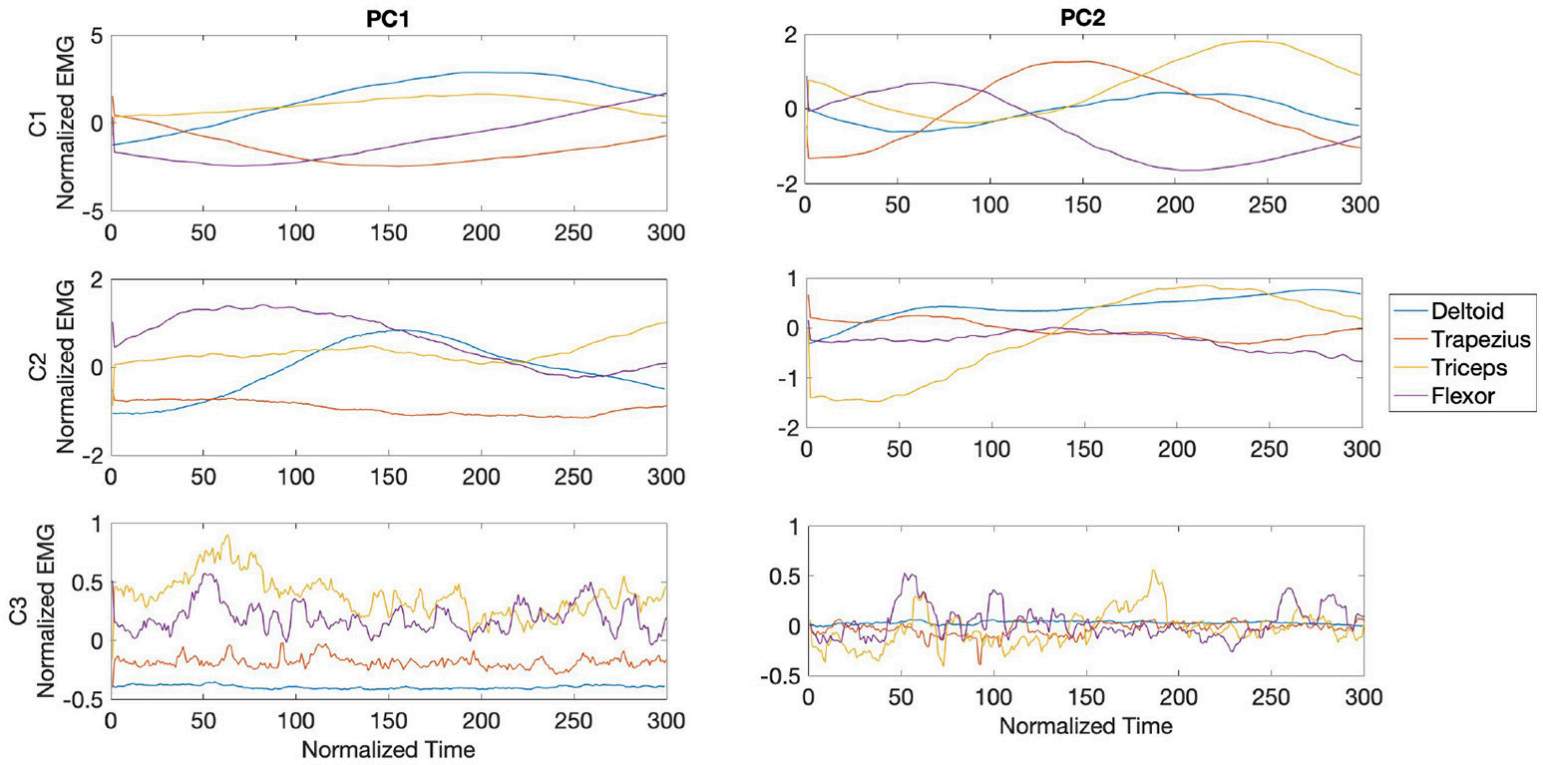
Waveforms showing the two principal components of muscle activation across the deltoid (blue), trapezius (orange), triceps (yellow), and wrist flexor (purple) muscles for cellists C1-3 in the fast phase (top panel in Figure 8). Each panel shows the activation pattern centered around bow onset (150 ms).

PC waveforms from participants C1 and C2, shown in Figure 12, exhibited consistent activity patterns from the deltoid in PC1, starting at the middle of the stroke. For all three participants PC1 was characterized by bursts from triceps, with activity from participant C3 made of consecutive high frequency bursts. PC2 in participant C1 was dominated by bursts from trapezius in the middle section of the bowing stroke, while triceps was the dominant muscle toward the end of the stroke for both participants C1 and C2.

**Figure 13:**
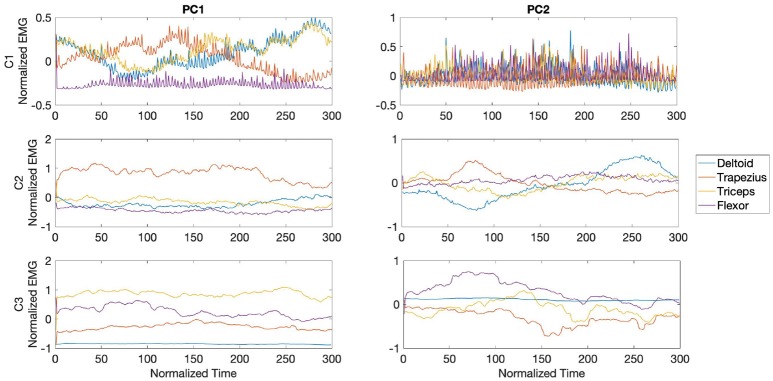
Waveforms showing the two principal components of muscle activation across the deltoid (blue), trapezius (orange), triceps (yellow), and wrist flexor (purple) muscles for cellists C1-3 in the transition phase (middle panel in Figure 8). Each panel shows the activation pattern centered around bow onset (150 ms).

During the transition phase, PC waveforms across cellists showed consistent patterns from participants C2 and C3, but distinctive high frequency bursts from participant C1. PC1 from C1 was characterized by bursts from trapezius in peaking at the center of the stroke, followed by bursts from triceps and deltoid as seen in Figure 13. Participants C2 and C3 exhibited dominant muscle activity from trapezius and from triceps respectively in PC1. PC2 from C1 displayed high frequency activity from all four muscles under analysis, while waveforms from participant C2 showed alternating bursts from trapezius and deltoid. Unlike participants C1 and C2, muscle activity patterns from participant C3 in PC2 are dominated by flexor bursts.

**Figure 14:**
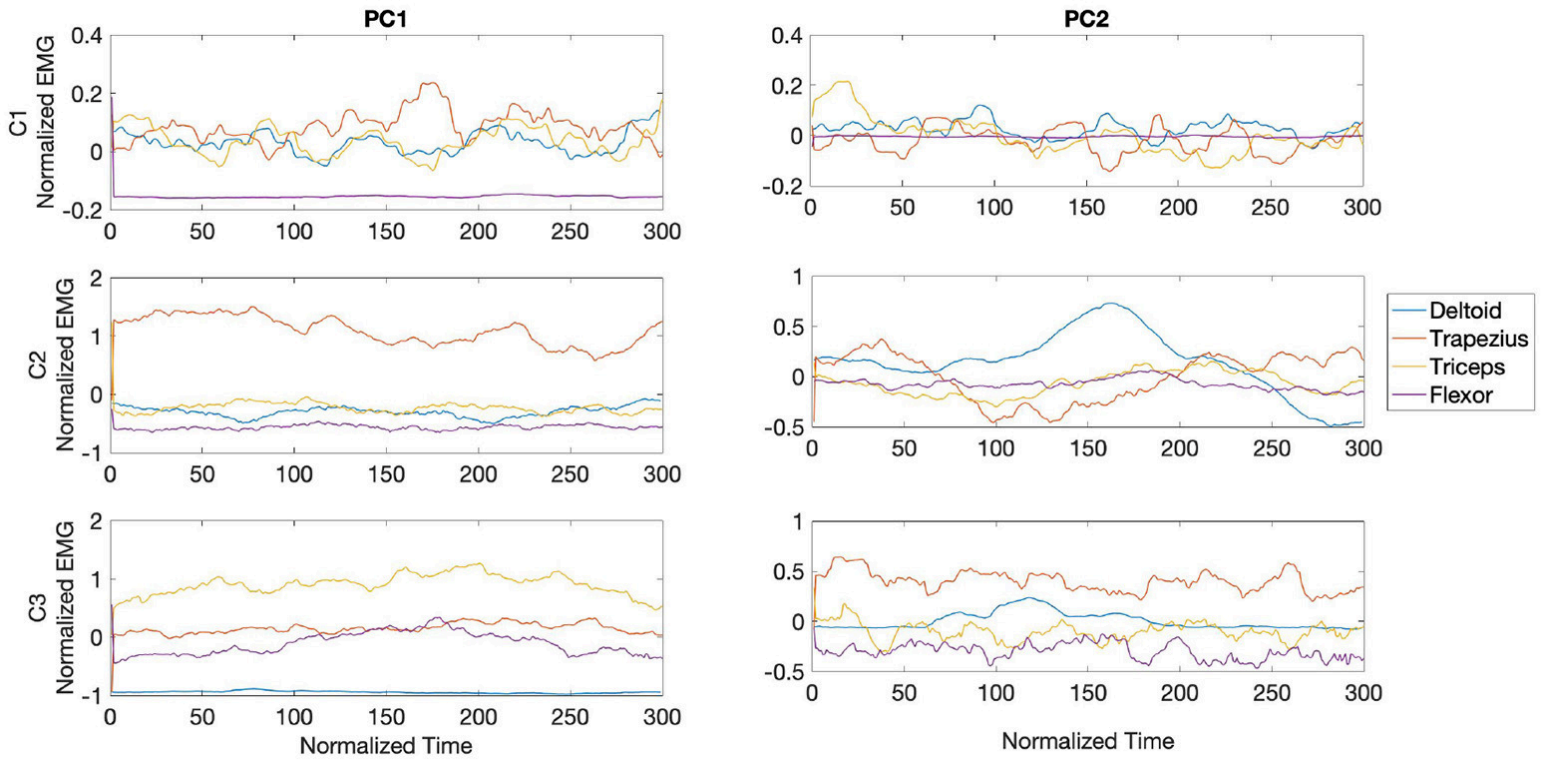
Waveforms showing the two principal components of muscle activation across the deltoid (blue), trapezius (orange), triceps (yellow), and wrist flexor (purple) muscles for cellists C1-3 in the slow phase (bottom panel in Figure 8). Each panel shows the activation pattern centered around bow onset (150 ms).

Figure 14 shows the waveforms from PC1 and PC2 for all three cellists during the slow phase. A distinct burst from trapezius around 150 ms characterized PC1 from participant C1. Participants C2 and C3 exhibited regular activity across the bowing stroke in PC1 from trapezius and triceps, respectively. Waveforms from PC2 are characterized by deltoid bursts in all three cellists, with participant C2 exhibiting a dominant deltoid burst toward the middle section of the stroke. A sequence of deltoid and trapezius bursts characterized PC2 from participant C1. Interestingly, variability in flexor activity is observed only from participant C3.

### 6.3. Contribution of Smoothness and Coarticulation

After investigating the individual components of fluency separately and for each instrument, we proceeded to explore how they combine to explain musical performance skill. Assuming that movement fluency is an integral part of expert music performance, we therefore propose a model where musical skill, as measured by the OMSI score, is predicted by movement smoothness (SAL) and coarticulation (number of PCs contributing to EMG variance).

Acknowledging the limited sample in our study, we include the a predictor for effects related to individual player and performed a multiple regression analysis for each phase, using the model

$$OMSI=\alpha +\beta_{1}Player+\beta_{2}SAL+\beta_{3}\#PC$$

where *OMSI* is the measure of musical sophistication, α is the intercept coefficient, and β_1_−β_3_ weights for the predictors *Player* (player), *SAL* (median SAL across strokes), and *#PC* (number of PCs for 60%).

The best fitting equations for the different playing phases were*Slow*: Predicted *OMSI*_*slow*_ = 1379.4–129.13 *Player* −22.28 *SAL* −168.42 *#PC*, [*F*_(3, 2)_ = 2.110, *p* = 0.34], with an adjusted *R*^2^ of 0.40.

*Transition*: Predicted *OMSI*_*trans*_ = −489.1 to −410.49 *Player* −673.60*SAL* −90.21*#PC*, [*F*_(3, 2)_ = 23.430, *p* = 0.04], with an adjusted *R*^2^ of 0.93.

*Fast*: Predicted *OMSI*_*fast*_ = −4119.8 to −684.80*Player* −2304.41 *SAL* −107.50 *#PC*, [*F*_(3, 2)_ = 1.459, *p* = 0.43], with an adjusted *R*^2^ of 0.22.

Without correcting for multiple tests the equation for the transition phase would just be significant on the .05 level, but the predictions from the equations should be interpreted with general caution. An additional effect of the very limited sample size was non-normally distributed residuals for the slow and transition phases, making it impossible to generalize from the models.

## 7. Discussion

The objective of this investigation was to explore the quantification of fluency as a combination of coordination and smoothness for drumming and cello bowing at different tempi. We therefore recorded movement kinematics and muscle activation data for a restricted sample of musicians of differing skill, which we assessed via the Ollen Musical Sophistication Index (OMSI). Arguably, the sample size is very small but serves as a proof of concept. The results show some promise and indicate that the approach can be useful to assess movement performance and generalize across players of different instruments and skill. Something that makes the approach attractive for use in technology assisted instrument pedagogy. In the following we will discuss our results along with some of the limitations of the study.

Our choices for measures of coordination and smoothness included motion capture and recording of muscle activity in a laboratory setting. One possible objection could be that currently these tools are not normally available for use by a music student during practice. While the development of an actual system for computer assisted tutoring is outside the scope of this paper, we argue that soon it will indeed be possible for such systems to record surface EMG as well as movement information. The rapid development of real-time tools for image processing (e.g., EyesWeb^3^ and OpenPose^4^), as well as the decreasing price of sensors (e.g., Myo^5^) makes the outlook for kinematic and EMG supported feedback very promising.

Some other methodological choices deserve discussion. We divided and analyzed data according to the temporal phases in the tempo acceleration - deceleration task which we identified as slow, transition, fast, and very fast. These playability areas for each player in Figure 3 illustrate how peak velocity varies with phase for each player. While the transition phase show clear changes in peak velocity for some players, the distinction is less clear for others. The critical points, identified by Visentin et al. (2008) as separating the phases of motor control for violinists, were distinguished by combined changes in both bowing distance, speed, and acceleration for professional players. In our study, the same break points can be discerned for player C1, but not for the less trained players.

Figure 3 also shows how the number of strokes available for analysis are unevenly distributed across the different phases. While the skilled players were able to play at very fast tempi, something the less trained students were unable to do, the slow phase did not always contain enough strokes. Specifically, player C1 only produced three strokes in total in before moving to the transition phase. This uneven distribution limited our options for statistical analysis. In particular, statistical comparisons between participants must be observed with caution. Kruskal–Wallis test comparisons were performed due to the non-normal distribution of data, however, the dependent nature of consecutive drumming and bowing strokes make it difficult to argue for independence of data points. Furthermore, in order to have enough data for the PCA for all players and phases, we included both up- and down strokes, increasing the number of data points. This limits the interpretation of the PCA waveforms for the cello strokes. To avoid similar problems in future studies, larger samples of participants should be instructed to remain at the slow, comfortable tempo until a minimum number of strokes have been collected before commencing the accelerando.

### 7.1. Movement Smoothness

In this study, we applied spectral arc length (SAL) as a metric for movement smoothness for strokes in drumming and cello. Spectral arc length has been tested against previously developed smoothness metrics, mostly aiming at clinical applications, evaluating hand movement from stroke and healthy individuals. Although our application of the metric differs somewhat compared to earlier studies, SAL has been proved to have superior sensitivity to changes in movement and reduced noise levels compared to previously developed jerk-based and peak metrics. Moreover, it was originally thought to be used to infer not only motor recovery, but also motor learning (Balasubramanian et al., 2012). Specifically, we here use SAL to compare movement smoothness between musicians of varying expertise across different instruments and movement duration. In this sense, the measure is here used for healthy movements where the velocity profile is expected to vary with tempo, and the results are expected to reflect expertise across movement temporal phases.

In the drumming task, SAL was shown to be mostly consistent across participants and phases, with the largest differences occurring during the slow phase. Drummers were shown to increase stroke smoothness at faster tempi and decrease smoothness at slow tempi. This is in correspondence with the higher peak acceleration for strokes at slow tempi reported by Dahl et al. (2011). Notably, for the slow phase participant D2 exhibited the lowest SAL values across all participants (see Figure 5), perhaps consistent with the lower musical sophistication index for this player (58, lowest OMSI score among drummers). Similarly, smoothness analysis of the cello task revealed consistent SAL values during the fast and transition phases, and larger differences between participants during the slow phase. Differences in OMSI score between participants seemed to be highlighted during the slow playing phase, as participant C1 exhibited the smoothest strokes when compared with C2 and C3. However, the number of strokes during the slow phase are too few to generalize.

A relationship between SAL and OMSI score during the transition and slow playing phases could be an indication of the degree of difficulty associated with accelerando sequences and the nature of the experiment, with participants concentrated on achieving maximum tempo even during the slower phases of the exercises. In particular, less experienced cellists were shown to be less able to properly shift between phases since their control system is not yet ready for performing fast bowing movements due to the lack of repetition and practice of such movements over time. Research within deliberate practice finds that virtuoso movements/advanced technical skills require vast and varied repetition over time. This is shown on fMRI samples from violinists and non-violinists (Schwenkreis et al., 2007). Furthermore, *expert* participants seemed to maintain a consistent movement smoothness across phases, while less experienced participants showed greater variability. These assumptions are in line with conclusions by Kerr et al. (2013), where hesitation in whole body movement (measured as drop in measured initial velocity) and smoothness were proposed as underlying factors of movement fluency. Following findings in Kerr et al. (2013), we could argue that the predicting power of smoothness in a fluency model during slow and transition phases will in part be linked to greater hesitation from less experienced players. Moreover, measures of smoothness have been shown to accurately reflect the ability of a performer on a specific task, assessing skillful motor control and execution with high sensitivity to familiarity to tasks and environments (Balasubramanian et al., 2015). In this sense, the use of smoothness toward skill prediction in our proposed model could lead to a more precise identification of proficiency development.

### 7.2. Muscle Coarticulation

We performed principal component analysis aiming at rendering additional evidence of variability of coarticulation patterns in expert and novice musicians across temporal phases and at using such coarticulation variability as input to a proposed model of fluency. Our results showed relatively consistent EMG patterns between cellists and drummers, with musicians with the highest OMSI score exhibiting less variability across temporal phases, and, in general, with fewer principal components accounting for the majority of the EMG variance.

While Winges et al. (2013) found that expert and amateur pianists show similar patterns of muscle activity to balance striking and non-striking digits while playing excerpts of musical pieces, we found differences across skill levels when musicians were asked to play tempo-changing exercises. Results from our study could be partially supported by findings by Parlitz et al. (1998), in which amateur pianists were shown to use greater force to achieve higher tempo when compared with professionals. In our study, variance in muscle activity in cellists with lower OMSI score was explained by a greater number of principal components during the transition and slow phases. On the other hand, PC patterns between drummers were less clear, although variability between phases was greater for participants with lower OMSI score.

In order to further examine patterns of coarticulation and their role in differences in skill levels between musicians and across temporal phases, we extracted the waveforms from each of the first two principal components for each participant. EMG patters from drummers in PC1 were relatively consistent in the fast and transition phases, with predominant bursts from trapezius alternated with bursts from triceps for all participants. Interestingly, bursts from participants D1 and D3 were shown to scale down as tempo decreased, while this trend is not observable in participant D2. These results could be supported by previous studies that have found coarticulation to be largely dependent on the duration and rate scaling of movement events, with muscles anticipating subsequent movement events (Sosnik et al., 2004; Klein Breteler et al., 2006; Winges et al., 2013; Godøy et al., 2017).

The variability in EMG patterns across cellists and across phases could be attributed to traces of such shaping of muscle contributions to adapt to specific goals, with waveforms in PC1 from participant C1 showing deltoid and triceps bursts scaling down in width as tempo decreased. In particular, PC1 during the transition phase displayed phasic coactivation of trapezius, triceps, and deltoid, while transition waveforms from participants C2 and C3 were mostly consistent activity from one muscle. These observed phasic muscular coactivation has been shown to modulate joint stiffness during time-varying force production tasks (Franklin and Wolpert, 2011; Winges et al., 2013), as was the case for the transition phase in our study. Moreover, the observed high frequency phasic coactivation from participant C3 during the fast phase might suggest joint stiffness partly due to lack of skill and experience. The coactivation for participant C1 in the fast phase was considerably smaller.

For our model of fluency, we selected the number of PCs required to explain 60% of variance but other measures of muscle activity that distinguish experts from novice performers are available. For instance Fujii et al. (2009b) measured the activity of agonist and antagonist muscles (flexor and extensor) for drummers and non-drummers and contrasted onset and offset times for bursts as well as co-contraction. Rather than focusing on agonist and antagonist muscles, we were interested to study the muscle activation from proximal to distal. Related theories on muscle modularity have proposed that bursts of fixed duration from multiple muscles make up synergistic control during complex sustained human movement, while variations in tempo have been shown to be related to scaling of the duration of EMG bursts (Flanders, 2002; d'Avella, 2016). Future research could therefore include the assessment of bursts timing.

### 7.3. Fluency

We aimed at exploring movement fluency not as a subjective measure of skill and function, but as an objective metric that can be measured through its underlying components. We speculated that the characteristics commonly attributed to fluent movements (such as graceful and effortless movement execution) can be assessed from the measurement of movement and motor control patterns in relation with music playing skill. Consequently, we used a measure of music sophistication (OMSI, Ollen, 2006) as our reference for musical performance skill, and computed linear regression models explaining OMSI as a function of smoothness and coarticulation. several studies have developed and validated objective measures of skill and movement quality, mainly based on kinematics, whereas fewer attempts have been made toward developing a robust and encompassing metric of movement fluency that takes into consideration other aspects of human movement and skill acquisition. Our proposed approach builds on efforts by Kerr et al. (2013), individually measuring aspects associated with movement fluency, although aiming at skill acquisition, performance, and assisted learning. As explored through regression analysis, the combination of SAL and number of PCs could contribute to explain some of the variability in the musical sophistication index, as measured by OMSI. However, due to the small sample size and the unbalanced number of observations between participants, the model should be interpreted with caution. A larger group of participants of different musical sophistication would allow the testing of a more robust and generalizable model and presumably also exclude “Player” as a predictor. Despite the limited number of participants in our study, we consider the span (46–984) in OMSI satisfactory. OMSI>500 indicates a high probability that the player would be rated as having high musical sophistication by an expert. Players C1 (984) and D1 (755) achieve high OMSI, which, together with their roles as professional instrumental teachers, support their status as “experts” in our study. However, we here make the assumption that the musical sophistication measured by OMSI is indicative of movement fluency. Although our assumption appears likely, based on the research reported in section 3, it remains an assumption without qualitative ratings of movement fluency directly, e.g., from point light representations of movement data. Such ratings could accompany a future study including more participants.

## 8. Conclusion

In our present exploratory study, we characterized fluency in music performance as a combination of movement smoothness and coarticulation, reflecting effort minimization through coordination strategies. Analysis of kinematic and muscle activation data of cello players and drummers of varying skill served as a proof of concept that the approach can be used for different kinds of movements, valuable for generic use in technology assisted music pedagogy. Although lacking the robustness and statistical validity to render conclusive evidence, the suggested model predicting a musical sophistication index (OMSI) can be further validated in future studies involving more players.

While a number of studies have investigated specific features of skilled movement in music performance, there are still open questions regarding individual differences due to anatomical and technical variance. We therefore see our contribution as a first step to objectively quantify a phenomenon that for years has been considered subjective and observational. We make use of measures and analysis techniques that have been shown to inform on performance related features, and discussed on the relevance of such features in order to fully understand the skill acquisition process in musical training. Furthermore, the analysis of sound producing actions might inherently require investigating the properties of the produced sound in order to fully assess the complex nature of music performance. Future studies will aim at the statistical validation of the proposed features and models of fluency in music performance, overcoming some of the methodological limitations of the present work, as well as diversifying instruments, techniques, and expertise levels.

## Author Contributions

RG conceived of the study. RG, VG-S, SD, and JH designed the experiment, adjusted and approved of the final manuscript. JH designed the cello exercises and recruited cellists. VG-S was main responsible for motion capture recording and main analysis of data. SD and VG-S were main responsible for writing the article.

### Conflict of Interest Statement

The authors declare that the research was conducted in the absence of any commercial or financial relationships that could be construed as a potential conflict of interest.

## Footnotes

^1^marcs-survey.uws.edu.au/OMSI/

^2^seniam.org

^3^infomus.org/eyesweb_ita.php

^4^github.com/CMU-Perceptual-Computing-Lab/openpose

^5^https://support.getmyo.com/hc/en-us
