# Supplementary material for: Characterizing Movement Fluency in Musical Performance: Toward a Generic Measure for Technology Enhanced Learning
Source: Front Psychol. 2019 Feb 4;10:84. doi: 10.3389/fpsyg.2019.00084 (PMC6369163; doi:10.3389/fpsyg.2019.00084)
Supplement: Supplementary file 1 [file Data_Sheet_1.PDF]

Supplementary Material: Characterizing movement fluency in musical performance: Toward a generic measure for technology enhanced learning

1 KINEMATIC ANALYSIS

For both instrument movements we calculated the magnitude of the linear velocity as the first order differentiation of the x, y, and z position and applied a second order zero-phase Butterworth smoothing filter with a cutoff frequency of 0.2 Hz. The velocity profile was then calculated as the magnitude of the veloctiy components using the Euclidian distance:

$$v_{mag} = \sqrt{{v_x}^2 + {v_y}^2 + {v_z}^2}$$

Both these steps were done through the MoCap toolbox for Matlab (Burger and Toiviainen 2013).

We then applied a peak finding algorithm (findpeaks, available from Mathworks) to identify peaks in the velocity profile v_{mag} (See Figure S1). In brief, the algorithm uses a threshold setting in combination with a minimum distance to identify between local maxima in the input signal. A similar method was used in Dahl 2004. Visual inspection is needed at this stage as the values may need adjustment for each individual player.

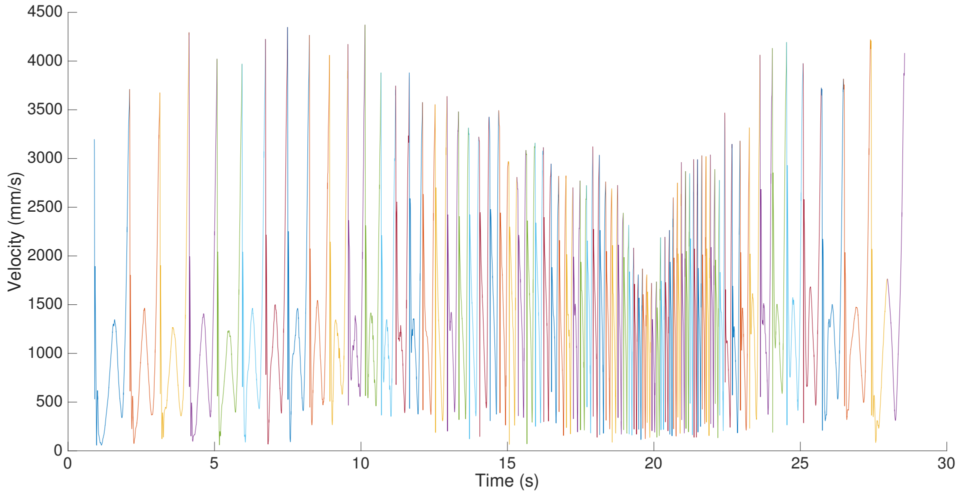

Figure S1. Segmented drum velocity profile from participant D1 during the single roll task.

After segmenting the signal with respect to the peak velocities we identified inter-onset intervals (ioi) for each stroke so that $ioi_1 = t_2 - t_1$, where ioi_1 is the time interval between the first and second stroke, with t_1 and t_2 the extracted time points for strokes 1 and 2 respectively.

Next, we obtained the spectral arc length (SAL) as the negative arc length of the velocity profiles' normalized Fourier magnitude spectrum (Balasubramanian et al. 2012; Rohrer et al. 2002; Balasubramanian et al. 2012, 2015):

$$SAL = -\int_0^{\omega_c} \sqrt{(\frac{1}{\omega_c})^2 + \left(\frac{dV(\omega)}{d\omega}\right)^2} d\omega \tag{S1}$$

where V(ω) represents the velocity profile's Fourier magnitude spectrum, while the range [0,ω_c] is the band frequency of the movement. For healthy human movements, the cut-off frequency used was 20 Hz (Balasubramanian et al. 2012). The algorithm to calculate the spectral arc length has been validated by and taken from Balasubramanian et al. (2012).

SAL was obtained for individual drum and cello strokes respectively, from segmented velocity profiles obtained as described above. A sequence of strokes' SAL metric was computed for each participant for each exercise and for each temporal phase.

2 PRINCIPAL COMPONENT ANALYSIS OF EMG

Muscle activity was recorded at 2000 Hz as surface EMG using Delsys Trigno (Boston, MA). An analogue trigger unit was used to synchronize both motion capture and EMG acquisitions in time.

Wireless EMG electrodes were placed on the location of the upper trapezius, triceps and forearm flexor for the drummers and, and deltoid, upper trapezius, triceps and forearm flexor for cellists, following suggestions from SENIAM [1].

The selection of muscles was based on previous studies on coarticulation of proximal and distal muscles during skillful tasks (Woollacott et al. 1986; Gandevia and Burke 1988; Shiratori and Latash 2000; Klein Breteler et al. 2006; Furuya and Kinoshita 2007; Verrel et al. 2013), while the decision to include deltoid data from cello players was based on previous findings on the importance of deltoid activity for appropriate playing technique in bow instruments (Ueno et al. 1998; Moss 2006; Verrel et al. 2013; Afsharipour et al. 2016).

Prior to time-varying principal component analysis, the EMG signal offset was removed from all collected data with a 50 Hz high pass filter. Data was then full-wave rectified by computing the absolute value of the raw signal. Finally, the EMG envelope was computed as the root mean square (RMS) value of the signal with a 25 ms moving window (De Luca 1997).

Smoothed EMG data was then segmented per stroke using the temporal locations of drum stick and bow strokes identified from peaks in the velocity profiles (as described in the previous section). The amplitude for each EMG segment was normalized according to the range of activity in the segment by subtracting the minimum value from that muscle and dividing by the maximum, resulting in muscle activity values between 0 and 1. Subsequently, the segmented EMG signals from each stroke were time normalized (resampling to 300 data points per stroke).

The input for the time-varying PCA for each participant was the smoothed, time-normalized EMG signal for each muscle for ***n*** number of strokes per temporal phase. The input matrix was then made of n stroke

[1] seniam.org

vectors x 3 EMG waveforms for the drumming tasks, and *n* stroke vectors x 4 EMG waveforms for the cello tasks (Figure S2).

$$EMG_{stroke} = meanEMG_{stroke} + PC1 \times EMG1_{stroke} + ... + PCn \times EMGn_{stroke} \qquad \text{(S2)}$$

The PCA results in a number of principal components (PCs) which are ranked in terms of the amount of variance in the data explained by each component (see Santello et al. 2002), revealing the main activation waveforms from the muscles under analysis, linked by their contribution to each stroke (Figure S2).

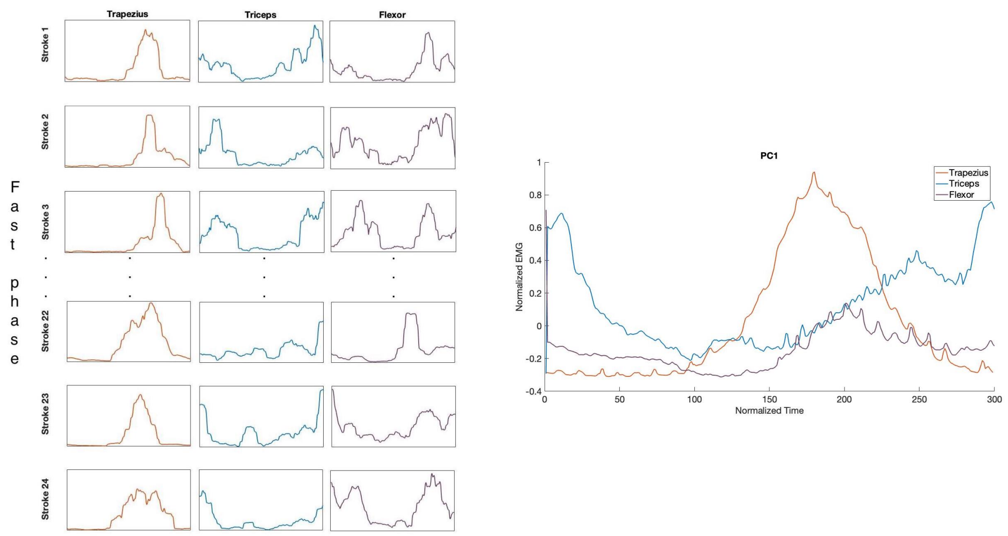

Figure S2. Left: An example of an input matrix for the time-varying principal component analysis. With *n* stroke EMG vectors as the input (stroke *1*. . . stroke *n*) per phase. **Right**: PC1 is shown with the color-coded normalized waveforms for the 3 muscles under analysis for the drumming tasks. The PCA results in a number of principal components (PCs) which are ranked in terms of the amount of variance in the data explained by each component. Each stroke can then be computed as its mean EMG amplitude plus the sum of the ranked PCs (with PC1 explaining the most variance, PC2 the second most variance etc). Data from participant D1.

REFERENCES

Afsharipour, B., Petracca, F., Gasparini, M., and Merletti, R. (2016). Spatial distribution of surface emg on trapezius and lumbar muscles of violin and cello players in single note playing. *Journal of Electromyography and Kinesiology* 31, 144–153

Balasubramanian, S., Melendez-Calderon, A., and Burdet, E. (2012). A robust and sensitive metric for quantifying movement smoothness. *IEEE transactions on biomedical engineering* 59, 2126–2136

Balasubramanian, S., Melendez-Calderon, A., Roby-Brami, A., and Burdet, E. (2015). On the analysis of movement smoothness. *Journal of neuroengineering and rehabilitation* 12, 112

Burger, B. and Toiviainen, P. (2013). MoCap Toolbox – A Matlab toolbox for computational analysis of movement data. In *Proceedings of the Sound and Music Computing Conference, Stockholm, Sweden* (KTH Royal Institute of Technology)

Dahl, S. (2004). Playing the accent - comparing striking velocity and timing in an ostinato rhythm performed by four drummers. *Acta Acustica united with Acustica* 90, 762–776

De Luca, C. J. (1997). The use of surface electromyography in biomechanics. *Journal of applied biomechanics* 13, 135–163

Furuya, S. and Kinoshita, H. (2007). Roles of proximal-to-distal sequential organization of the upper limb segments in striking the keys by expert pianists. *Neuroscience letters* 421, 264–269

Gandevia, S. and Burke, D. (1988). Projection to the cerebral cortex from proximal and distal muscles in the human upper limb. *Brain* 111, 389–403

Klein Breteler, M. D., Simura, K. J., and Flanders, M. (2006). Timing of muscle activation in a hand movement sequence. *Cerebral Cortex* 17, 803–815

Moss, K. D. (2006). *Favored Sound Production Exercises of Selected Violin, Viola, Cello, and Double Bass Pedagogues: An Analysis and Adaptation*. Ph.D. thesis, University of Florida

Rohrer, B., Fasoli, S., Krebs, H. I., Hughes, R., Volpe, B., Frontera, W. R., et al. (2002). Movement smoothness changes during stroke recovery. *Journal of Neuroscience* 22, 8297–8304

Santello, M., Flanders, M., and Soechting, J. F. (2002). Patterns of hand motion during grasping and the influence of sensory guidance. *Journal of Neuroscience* 22, 1426–1435

Shiratori, T. and Latash, M. (2000). The roles of proximal and distal muscles in anticipatory postural adjustments under asymmetrical perturbations and during standing on rollerskates. *Clinical neurophysiology* 111, 613–623

Ueno, K., Frukawa, K., Nagano, M., Asami, T., Yoshida, R., Yoshida, F., et al. (1998). Good posture improves cello performance. In *Engineering in Medicine and Biology Society, 1998. Proceedings of the 20th Annual International Conference of the IEEE* (IEEE), vol. 5, 2386–2389

Verrel, J., Pologe, S., Manselle, W., Lindenberger, U., and Woollacott, M. (2013). Exploiting biomechanical degrees of freedom for fast and accurate changes in movement direction: coordination underlying quick bow reversals during continuous cello bowing. *Frontiers in human neuroscience* 7, 157

Woollacott, M. H., Shumway-Cook, A., and Nashner, L. M. (1986). Aging and posture control: changes in sensory organization and muscular coordination. *The International Journal of Aging and Human Development* 23, 97–114
